# Supplementary material for: Predator-secreted sulfolipids induce defensive responses in C. elegans
Source: Nat Commun. 2018 Mar 19;9:1128. doi: 10.1038/s41467-018-03333-6 (PMC5859177; doi:10.1038/s41467-018-03333-6)

Predator-secreted sulfolipids induce defensive responses in *C. elegans*

Zheng Liu[1]†, Maro J. Kariya[2]†, Christopher D. Chute[3]†, Amy K. Pribadi[1,4], Sarah G. Leinwand[1], Ada Tong[1], Kevin P. Curran[1], Neelanjan Bose[2], Frank C. Schroeder[2], Jagan Srinivasan[3] and Sreekanth H. Chalasani[1,4,5]

[1] Molecular Neurobiology Laboratory, The Salk Institute for Biological Studies, La Jolla, CA 92037.

[2] Boyce Thompson Institute and Department of Chemistry and Chemical Biology, Cornell University, Ithaca, NY 14853.

[3] Department of Biology and Biotechnology, Worcester Polytechnic Institute, Worcester, MA 01605.

[4] Division of Biological Sciences, University of California, San Diego, La Jolla, CA 92093.

† These authors contributed equally to this work.

[5] Correspondence to: (S.H.C.) schalasani@salk.edu

Table of Contents

1. Supplementary Figures S1-S6

2. Supplementary Tables S1-S8

3. Supplementary Methods (NMR spectra)

Supplementary Figures

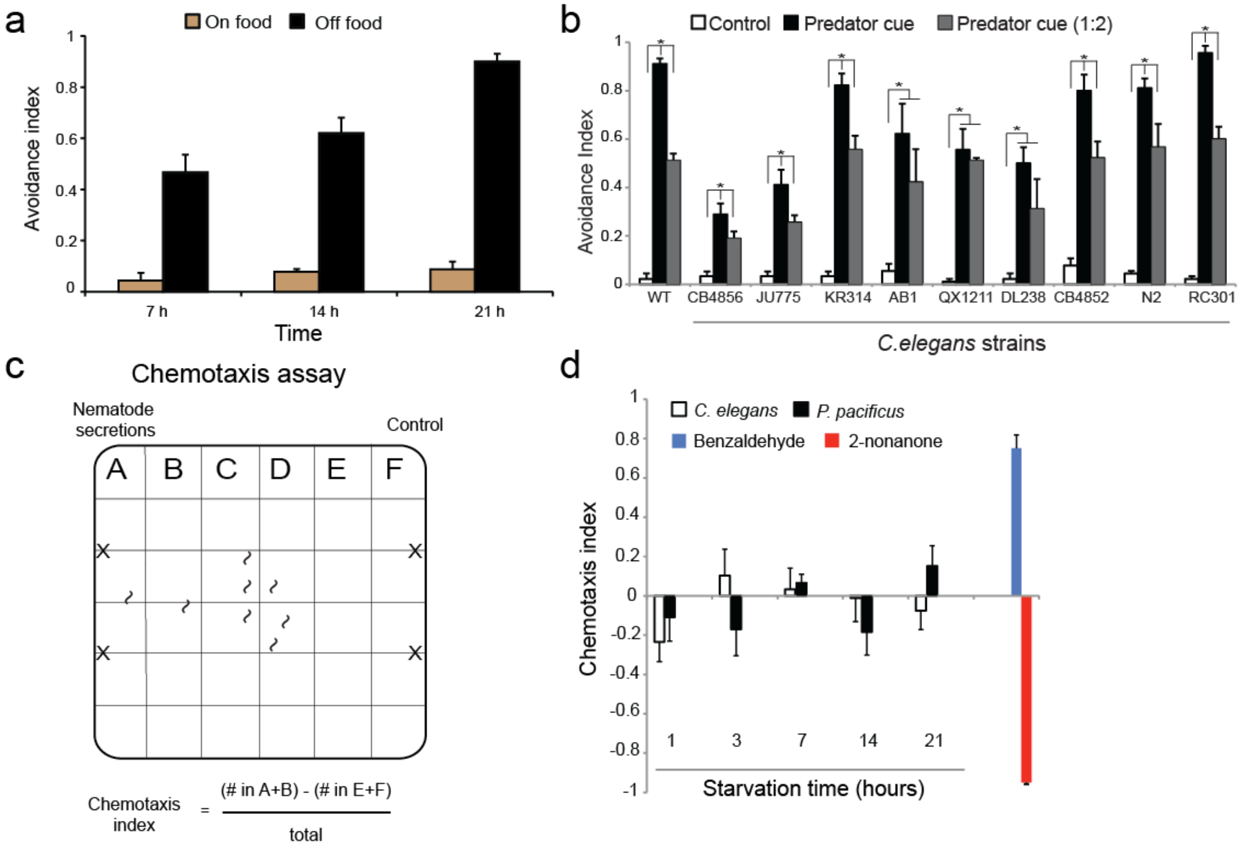

Supplementary Figure S1. *C. elegans* responds to a water soluble, but not volatile component of predator secretions. a, *C. elegans* shows strong avoidance to secretions collected from starving, but not well-fed, *Pristionchus pacificus* (RS5275B). **b**, Avoidance responses of multiple *C. elegans* isolates to Control and predator cue: CB4852, N2 (Oxford, England), AB1 (from Adelaide, Australia), QX1211 (from San Francisco, US), DL238 (from Hawaii, USA), JU775 (Paris, France) and CB4856 (from Hawaii, USA). **c**, Schematic showing the chemotaxis assay used to measure *C. elegans* responses to volatile components. Nematode secretions were placed in column A, with control (M9 buffer) in column F at the regions marked by ‘x’. Animals were placed in column C and D allowed to chemotax for 60 min and were scored as indicated. **d,** *C. elegans* was not attracted to or repelled by secretions collected from starving nematodes for different durations. In contrast, they showed strong responses to a known attractant, Benzaldehyde (1:200), and the repellent, 2-nonanone (undiluted). n =12, averages and s.e.m. are shown and * indicates $p < 0.05$ fisher’s exact t-test with Bonferroni correction.

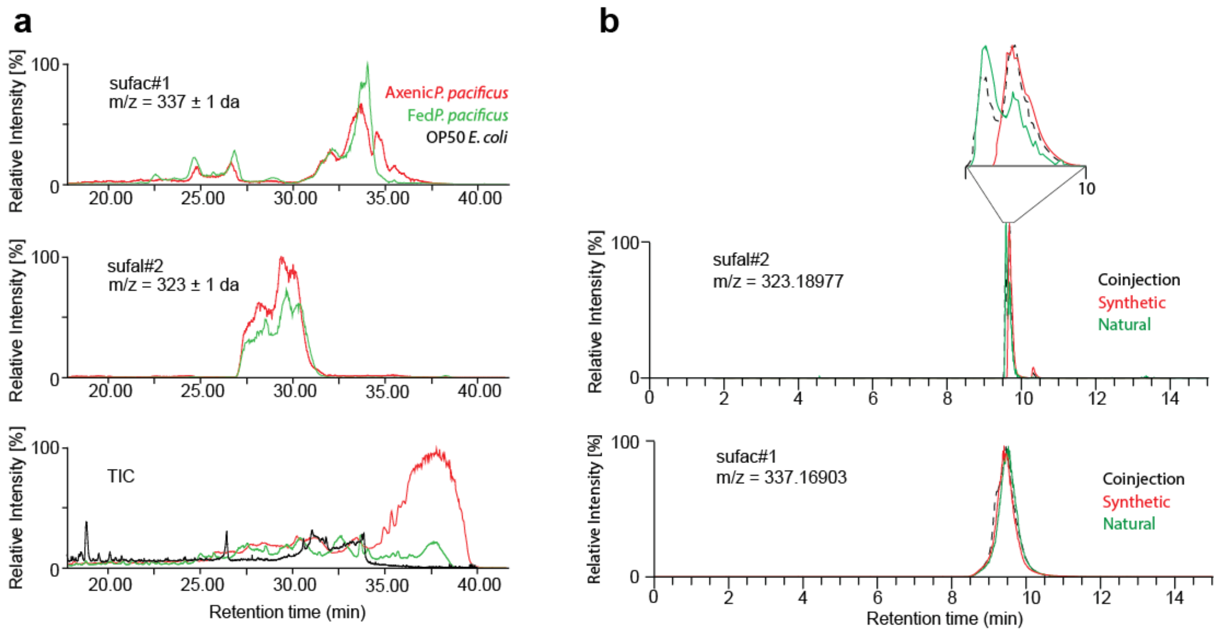

Supplementary Figure S2. Properties of *P. pacificus* sulfates. a, HPLC-MS analysis of exo-metabolome extracts from *P. pacificus* under fed (green) and axenic (red) conditions and an extract from OP50 *E. coli* (black). Traces for sufac#1 and sufal#2 are shown in the upper panels and the total ion chromatogram (TIC) in the lower panel (all intensities are normalized to pasc#9 intensity). Both fed and axenic cultures produced sufac#1 and sufal#2. **b**, UHPLC-HRMS analysis of natural (green), synthetic (red), and natural/synthetic-coinjected sufac#1 and sufal#2 (black-dotted).

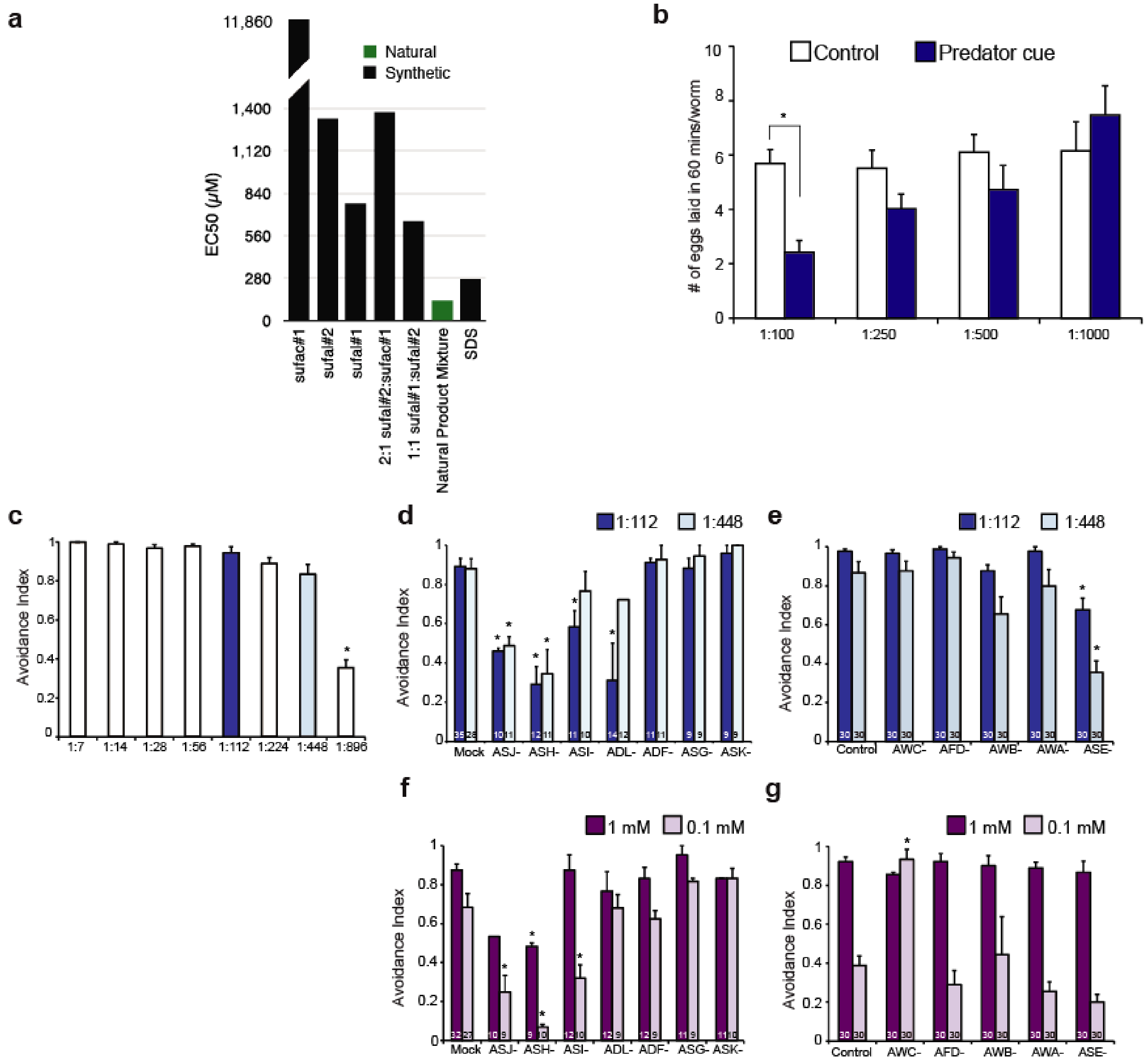

Supplementary Figure S3: *C. elegans* responses to sulfates. a, EC50 concentrations for *C. elegans* avoidance responses to synthetic sulfates and sulfate mixtures, compared to avoidance responses to a mixture of sulfates isolated from *P. pacificus* exo-metabolome (for data used to calculate EC50, see Supplementary Table S6). **b**, Sulfolipid pre-exposure transiently attenuates egg laying behavior. Animals were pre-exposed to predator cue or DMSO for 30 minutes and the number of eggs laid in the subsequent 60 minutes are shown. **c**, *C. elegans* shows a dose-dependent avoidance of the active sulfate-containing compounds. See Supplementary Table S6 for responses to control stimuli. Animals lacking ASJ, ASH, ASI, ADL, and ASE neurons are defective in their responses to active sulfates, as shown using **d**, cell and **e**, genetic ablations. In contrast, animals lacking ASI, ASH, and ASJ neurons are defective in their avoidance responses to SDS again shown using **f**, cell and **g**, genetic ablations Averages and s.e.m. are shown. Number of animals tested is 30 (**a**, **c**), > 38 (**b**) and indicated in (**d-g**). * indicates $p < 0.05$ comparing that strain or condition with corresponding controls obtained using Fisher's exact t-test with Bonferroni correction.

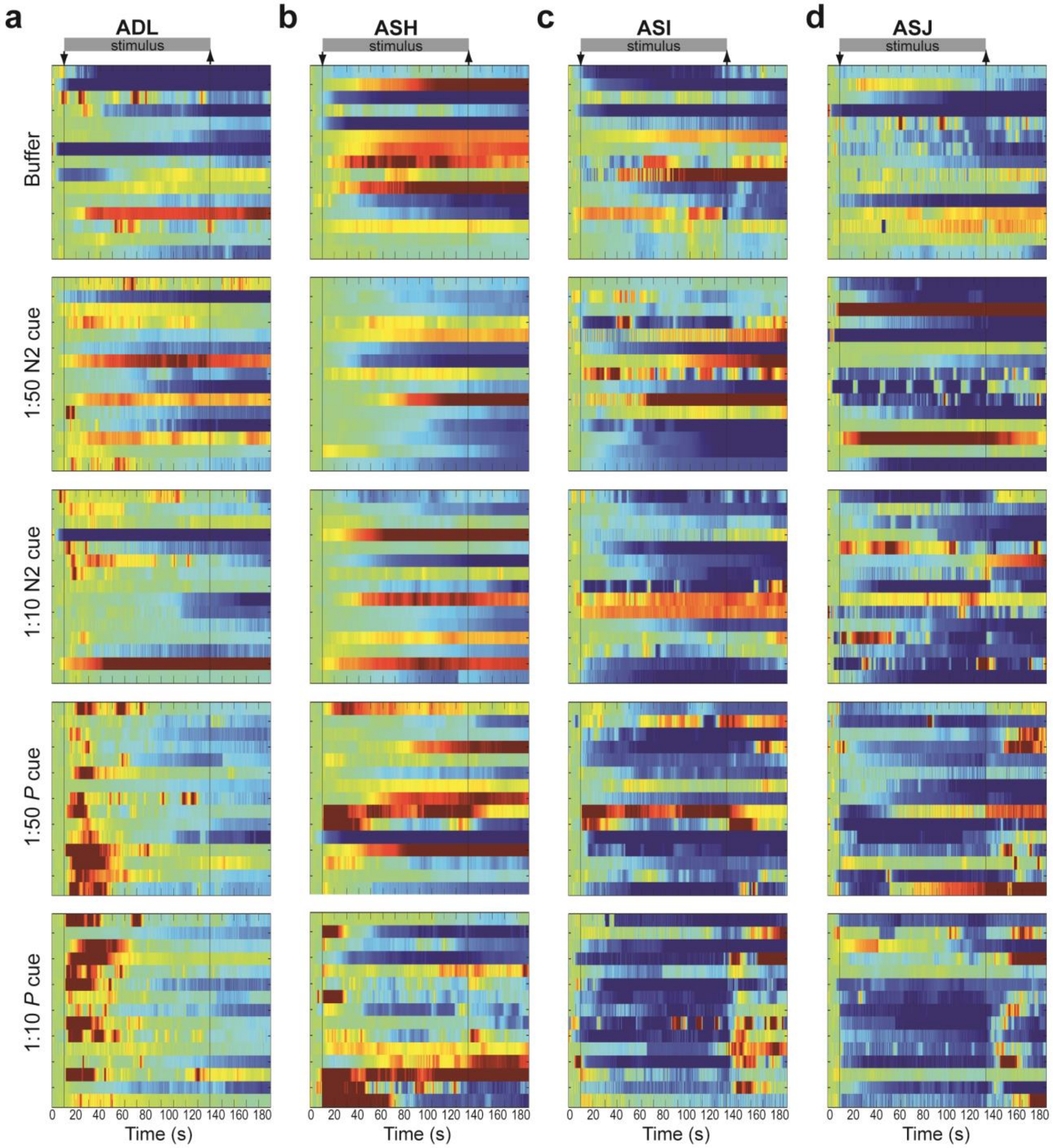

Supplementary Figure S4: Sensory neuron responses to nematode secretions. Heat maps showing **a**, ADL, **b**, ASH, **c**, ASI and **d**, ASJ responses to buffer, *C. elegans* secretions (N2 cue) and Predator cue (P cue) diluted 1:10 and 1:50. Each row represents responses from a single neuron in an animal, which was only stimulated once. Scale bars are shown on the right with warmer colors (larger ΔF/F) representing larger increases in neuronal activity. n = 13-15 for each condition.

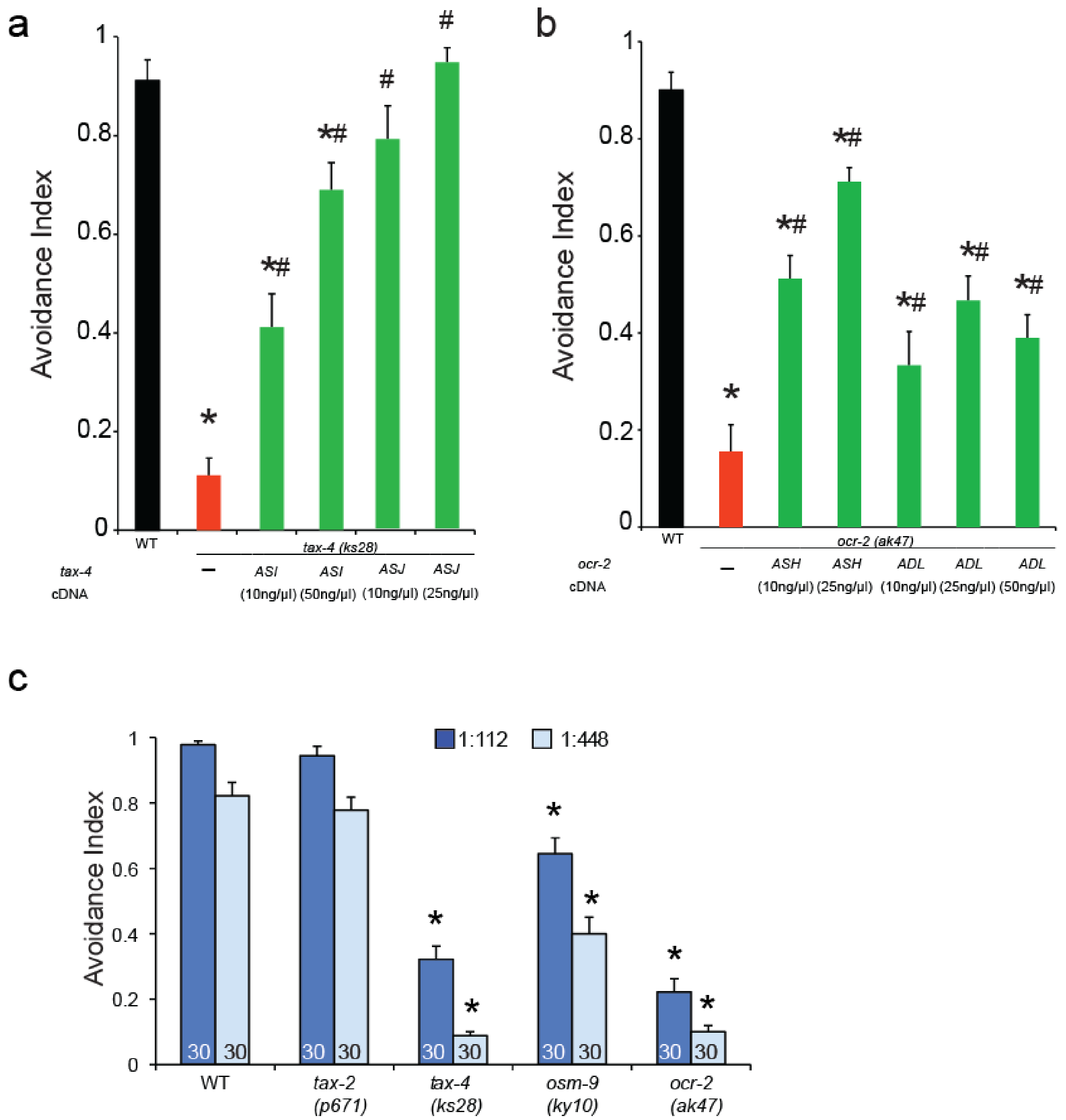

Supplementary Figure S5. Dose-dependent effects of *tax-4* and *ocr-2* transgenes. a, Mutants in *tax-4*, but not *tax-2* channel are defective in their response to predator cue. Restoring TAX-4 function to ASI or ASJ restores normal behavior to the *tax-4* null mutants in a dose-dependent manner. **b**, *ocr-2*, but not *osm-9* mutants are defective in their avoidance response to predator cue. Normal behavior is restored when *ocr-2* cDNA is expressed in either ASH or ADL in a dose dependent manner. **c**, Avoidance index of various mutant strains exposed to sulfolipids. * indicates $p < 0.05$ to wildtype, while # indicates $p < 0.05$ compared to mutants obtained using Fisher's exact t-test with Bonferroni correction.

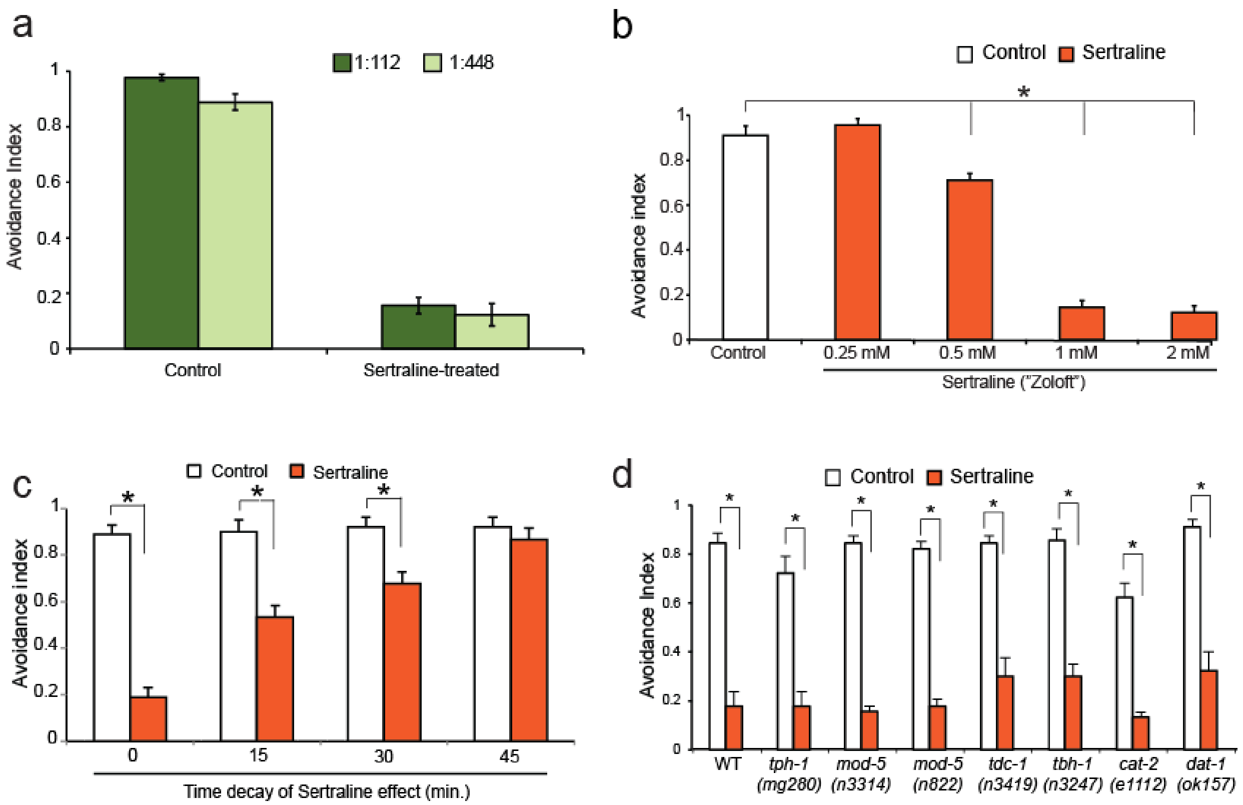

Supplementary Figure S6: Sertraline pre-treatment attenuates *C. elegans* avoidance responses to predator cue and sulfolipids. a, Sertraline attenuates avoidance behavior upon exposure to sulfolipids. **b,** Dose dependence of sertraline attenuation of predator avoidance. **c**, Time course of the decay of the sertraline effect. The drug effects last about 30 minutes. **d**, Sertraline does not require biogenic amine signaling to exert its effects on *C. elegans* avoidance behavior. Sertraline modulates avoidance responses of mutants in dopamine (*cat-2*, (catecholamine synthase required for dopamine synthesis and *dat-1*, dopamine transporter), serotonin (*tph-1*, tryptophan hydroxylase the rate limiting step in serotonin synthesis and *mod-5*, serotonin re-uptake transporter), octopamine *t dc-1* tyrosine decarboxylase required for synthesis of tyramine and octopamine), or tyramine signaling *tbh-1* (tyramine beta-hydroxylase required for tyramine synthesis). Data in **b, c,** and **d** show *C. elegans* responses to predator cue. Averages and s.e.m. are shown. n > 30 animals tested on at least 3 different days. * indicates $p < 0.05$ comparing that condition with the corresponding controls obtained using Fisher's exact t-test with Bonferroni correction.

2. Supplementary Tables

Supplementary Table S1. ^{1}H and ^{13}C NMR spectroscopic data for sufal#2 in methanol-d_4. Chemical shifts were referenced to δ($CD_2\underline{H}OD$) = 3.31 and δ($^{13}\underline{C}D_3OD$) = 49.0. ^{13}C chemical shifts were determined via HMBC and HSQC spectra. Spectra were acquired using the Bruker Avance 800 spectrometer. ^{1}H, ^{1}H-J-coupling constants were determined from the acquired ^{1}H or dqfCOSY spectra. HMBC correlations are from the proton(s) stated to the indicated ^{13}C atom.

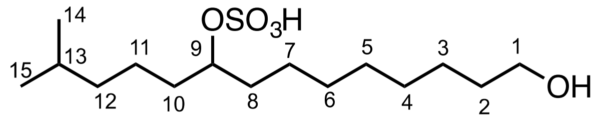

Position	^{13}C [ppm]	^{1}H [ppm]	^{1}H-^{1}H coupling constants (Hz)	HMBC correlations
1	62.76	1a = 3.53<br>1b = 3.53	$J_{1,2}$ = 6.80	2, 3
2	33.40	2a =1.53<br>2b = 1.53		1, 3, 4
3	26.64	3a = 1.34<br>3b = 1.34		1, 2, 4, 5
4	30.28	4a = 1.32<br>4b = 1.32		2, 3, 5, 6
5	30.32/<br>30.29	5a =1.32-1.35<br>5b =1.32-1.35		-
6	30.29/<br>30.32	6a =1.32-1.35<br>6b =1.32-1.35		-
7	25.75	7a = 1.41<br>7b = 1.41		8, 9
8	35.02	8a = 1.62<br>8b = 1.65		7, 9, 10
9	80.70	4.33	$J_{9,\ 8a\ 8b\ 10a\ 10b}$ = 6.13	7, 8, 10, 11
10	35.27	10a = 1.59<br>10b = 1.64		8, 9, 11, 12
11	23.59	11a = 1.39<br>11b = 1.43		9, 10, 12, 13,
12	39.94	12a = 1.18<br>12b = 1.22		10, 11, 13, 14
13	28.82	1.55		11, 12, 14, 15
14	22.74	0.89	$J_{14,13}$ = 6.36	12, 13, 15
15	22.74	0.89	$J_{15,13}$ = 6.36	12, 13, 14

Supplementary Table S2. ^{1}H and ^{13}C NMR spectroscopic data for sufac#1 in methanol-d_4. Chemical shifts were referenced to δ($CD_2\underline{H}OD$) = 3.31 and δ($^{13}\underline{C}D_3OD$) = 49.0. ^{13}C chemical shifts were determined via HMBC and HSQC spectra. Spectra were acquired using the Bruker Avance 800 spectrometer. ^{1}H, ^{1}H-J-coupling constants were determined from the acquired ^{1}H or dqfCOSY spectra. HMBC correlations are from the proton(s) stated to the indicated ^{13}C atom.

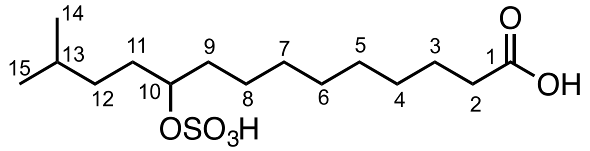

Position	^{13}C [ppm]	^{1}H [ppm]	^{1}H-^{1}H coupling constants (Hz)	HMBC correlations
1	177.40	---		-
2	34.70	2a = 2.28<br>2b = 2.28	$J_{2,3}$ = 7.55	1, 3, 4
3	25.83	3a = 1.60<br>3b = 1.60		1, 2, 4
4	29.92	4a = 1.34<br>4b = 1.34		-
5	30.09	5a = 1.35<br>5b = 1.35		3, 4
6	30.22/30.37	6a = 1.32-1.35<br>6b = 1.32-1.35		-
7	30.37/30.22	7a = 1.32-1.35<br>7b = 1.32-1.35		-
8	25.75	8a = 1.39<br>8b = 1.39		-
9	35	9a = 1.63<br>9b = 1.65		8, 10, 11
10	80.85	4.31	$J_{10,\ 9a\ 9b\ 11a\ 11b}$ = 6.15	8, 9, 11, 12
11	32.91	11a = 1.63<br>11b = 1.67		9, 10, 12, 13
12	34.87	12a = 1.26<br>12b = 1.31		10, 11, 13, 14, 15
13	28.98	1.54		11, 12, 14, 15
14	22.68	0.90	$J_{14,13}$ = 6.59	12, 13, 15
15	22.68	0.90	$J_{15,13}$ = 6.59	12, 13, 14

Supplementary Table S3. Comparison of ^{13}C NMR spectroscopic data for sufac#1 and sufal#2 in methanol-d$_4$. Chemical shifts were referenced $\delta(^{13}\underline{C}D_3OD) = 49.0$. ^{13}C chemical shifts were determined via HMBC and HSQC spectra for natural and 1D carbon for synthetic. Spectra were acquired using the Bruker Avance 800 spectrometer for natural and Varian INOVA-600 for synthetic samples.

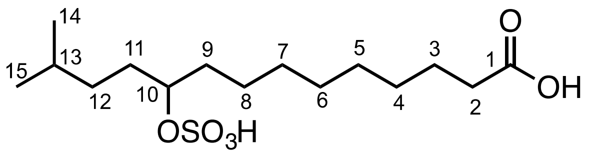

sufac#1			
Position	^{13}C Natural [ppm]	^{13}C Synthetic [ppm]	Δppm
1	177.40	178.46	-1.06
2	34.70	35.10	-0.40
3	25.83	26.31	-0.48
4	29.92	30.54	-0.62
5	30.09	30.73	-0.64
6	n/a	30.39	n/a
7	n/a	30.29	n/a
8	25.75	25.99	-0.24
9	35.00	35.56	-0.56
10	80.85	81.13	-0.28
11	32.91	33.09	-0.18
12	34.87	35.23	-0.36
13	28.98	29.26	-0.28
14	22.68	23.04	-0.36
15	22.68	22.96	-0.28

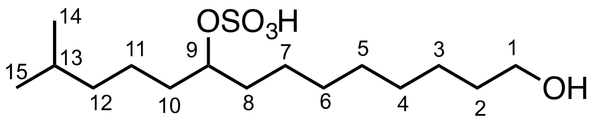

sufal#2			
Position	^{13}C Natural [ppm]	^{13}C Synthetic [ppm]	Δppm
1	62.76	63.03	-0.27
2	33.40	33.69	-0.29
3	26.64	26.93	-0.29
4	30.28	30.54	-0.26
5	n/a	30.74	n/a
6	n/a	30.67	n/a
7	25.75	26.03	-0.28
8	35.02	35.30	-0.28
9	80.70	81.04	-0.34
10	35.27	35.55	-0.28
11	23.59	23.87	-0.28
12	39.94	40.23	-0.29
13	28.82	29.11	-0.29
14	22.74	23.03	-0.29
15	22.74	23.03	-0.29

Supplementary Table S4. Nematode species analyzed by HPLC-MS for the presence of sulfated lipids.

Pratylenchus penetrans
Panagrellus redivivus
Pelodera strongyloides
Nippostrongylus brasiliensis (A)
Nippostrongylus brasiliensis (IJ)
Heterorhabditis bacteriophora
Oscheius tipulae
Oscheius carolinensis (A)
Oscheius carolinensis (IJ)
Caenorhabditis elegans
Caenorhabditis sp.7
Caenorhabditis sp.7 (dauer)
Rhabditis sp.
Ascaris suum
Pristionchus pacificus
Koernia sp.
Steinernema carpocapsae (A)
Steinernema carpocapsae (IJ)
Steinernema scapterisci (IJ)
Steinernema riobrave (IJ)
Steinernema glaseri (A)
Steinernema glaseri (IJ)
Romanomermis iyengari (A)
Romanomermis iyengari (IJ)
Romanomermis culicivorax (A)
Trichuris suis
Ancylostoma ceylanicum

Supplementary Table S5. Table showing drugs tested for their ability to attenuate *C. elegans* responses to *Predator cue*.

Strain	Drug	Stimulus	# of animals	Average A.I.	SEM	Statistics
N2	M9 control	Predator cue	30	0.91111	0.04006	
N2	Nortriptyline	Predator cue	30	0.87778	0.04006	
N2	Chlorpromazine	Predator cue	30	0.95556	0.01111	
N2	Thioridazine	Predator cue	30	0.90000	0.05092	
N2	Imipramine	Predator cue	30	0.91111	0.04006	
N2	Chlorprothixene	Predator cue	30	0.78889	0.04843	
N2	Triflupromazine	Predator cue	30	0.86667	0.03333	
N2	Propionylpromazine	Predator cue	30	0.90000	0.01925	
N2	Mianserin	Predator cue	30	0.64444	0.04843	$p < 0.05$ compared to control
N2	Cocaine	Predator cue	30	0.84444	0.04006	
N2	Fluoxetine	Predator cue	30	0.84444	0.02940	
N2	Sertraline	Predator cue	30	0.58889	0.05879	$p < 0.05$ compared to control
N2	DMSO	Predator cue	30	0.93333	0.03849	

Supplementary Table S6. Table showing the independence of avoidance assay

Repellent	Trial 1 Average ± 95% confidence interval	Trial 2 Average ± 95% confidence interval	Trial 3 Average ± 95% confidence interval
Predator cue	0.8 ± 0.12	0.875 ± 0.10325	0.825 ± 0.116
Predator cue - 1:10 dilution	0.4 ± 0.14525	0.375 ± 0.142375	0.575 ± 0.1465
1 mM SDS	0.96 ± 0.035	0.87 ± 0.091	0.96 ± 0.073
0.1 mM SDS	0.65 ± 0.41	0.7 ± 0.064	0.65 ± 0.087

Supplementary Table S7. Table showing avoidance indices of all strains tested and their controls.

Exp #	Strain	Genotype	Condition	Stimulus	# of test	Average A.I.	SEM	Stats	Figure
1	N2			*C. elegans* secretions after 1hr starvation	30 animals, each tested thrice	0.10000	0.01925		Fig. 1a
2	N2			*P. pacificus* secretions after 1hr starvation	30 animals, each tested thrice	0.10000	0.03333		Fig. 1a
3	N2			*C. elegans* secretions after 3hr starvation	30 animals, each tested thrice	0.11111	0.02940		Fig. 1a
4	N2			*P. pacificus* secretions after 3hr starvation	30 animals, each tested thrice	0.21111	0.02940		Fig. 1a
5	N2			*C. elegans* secretions after 7hr starvation	30 animals, each tested thrice	0.01111	0.01111		Fig. 1a
6	N2			*P. pacificus* secretions after 7hr starvation	30 animals, each tested thrice	0.37778	0.04843	significant compared to Exp#5	Fig. 1a
7	N2			*C. elegans* secretions after 14hr starvation	30 animals, each tested thrice	0.07778	0.02940		Fig. 1a
8	N2			*P. pacificus* secretions after 14hr starvation	30 animals, each tested thrice	0.61111	0.05879	significant compared to Exp#7	Fig. 1a
9	N2			*C. elegans* secretions after 21hr starvation	30 animals, each tested thrice	0.14444	0.02940		Fig. 1a
10	N2			*P. pacificus* secretions after 21hr starvation	30 animals, each tested thrice	0.66667	0.05092	significant compared to Exp#9	Fig. 1a
11	N2			*C. elegans* secretions after 21hr with food	30 animals, each tested thrice	0.00000	0.00000		Fig. 1a
12	N2			*P. pacificus* secretions after 21hr with food	30 animals, each tested thrice	0.02222	0.02222		Fig. 1a
13	N2			*P. pacificus* PS312 secretions after 1hr starvation	30 animals, each tested thrice	0.02222	0.01111		Fig. 1a
14	N2			*P. pacificus* RS5275B secretions after 1hr starvation	30 animals, each tested thrice	0.04444	0.01111		Fig. 1a
15	N2			*P. pacificus* PS312 secretions after 3hr	30 animals, each tested thrice	0.17778	0.01111		Fig. 1a

				starvation					
16	N2			*P. pacificus* RS5275B secretions after 3hr starvation	30 animals, each tested thrice	0.15555	0.04006		Fig. 1a
17	N2			*P. pacificus* PS312 secretions after 5hr starvation	30 animals, each tested thrice	0.24444	0.05879		Fig. 1a
18	N2			*P. pacificus* RS5275B secretions after 5hr starvation	30 animals, each tested thrice	0.32222	0.08012		Fig. 1a
19	N2			*P. pacificus* PS312 secretions after 7hr starvation	30 animals, each tested thrice	0.35556	0.07778		Fig. 1a
20	N2			*P. pacificus* RS5275B secretions after 7hr starvation	30 animals, each tested thrice	0.46667	0.06939		Fig. 1a
21	N2			*P. pacificus* PS312 secretions after 21hr starvation	30 animals, each tested thrice	0.61111	0.05879		Fig. 1a
22	N2			*P. pacificus* RS5275B secretions after 21hr starvation	30 animals, each tested thrice	0.90000	0.03849	significant compared to Exp#21	Fig. 1a
23	N2			*P. pacificus* PS312 secretions after 48hr starvation	30 animals, each tested thrice	0.12222	0.04843		Fig. 1a
24	N2			*P. pacificus* RS5275B secretions after 48hr starvation	30 animals, each tested thrice	0.13333	0.01925		Fig. 1a
25	Mock			M9 control	65 animals, each tested thrice	0.08056	0.02769		
26	ASJ-			M9 control	13 animals, each tested thrice	0.11852	0.06458		
27	ASH-			M9 control	10 animals, each tested thrice	0.05556	0.05556		
28	ASI-			M9 control	12 animals, each tested thrice	0.12778	0.07222		
29	ADL-			M9 control	13 animals, each tested thrice	0.12593	0.00741		
30	ADF-			M9 control	12 animals, each	0.07222	0.03889		

					tested thrice				
31	ASG-			M9 control	11 animals, each tested thrice	0.04167	0.04167		
32	ASK-			M9 control	7 animals, each tested thrice	0.13889	0.02778		
33	Mock			Predator cue	65 animals, each tested thrice	0.77361	0.04341		Fig. 2b
34	ASJ-			Predator cue	13 animals, each tested thrice	0.44074	0.13764	significant compared to Exp#33	Fig. 2b
35	ASH-			Predator cue	10 animals, each tested thrice	0.28333	0.02546	significant compared to Exp#33	Fig. 2b
36	ASI-			Predator cue	12 animals, each tested thrice	0.47963	0.15249	significant compared to Exp#33	Fig. 2b
37	ADL-			Predator cue	13 animals, each tested thrice	0.21481	0.05185	significant compared to Exp#33	Fig. 2b
38	ADF-			Predator cue	12 animals, each tested thrice	0.72407	0.10346		Fig. 2b
39	ASG-			Predator cue	11 animals, each tested thrice	0.64583	0.02083		Fig. 2b
40	ASK-			Predator cue	7 animals, each tested thrice	0.90278	0.01389		Fig. 2b
41	N2			M9 control	30 animals, each tested thrice	0.08889	0.01111		
42	PY7502 (AWC-)	*oyIs85*		M9 control	30 animals, each tested thrice	0.11111	0.02940		
43	PR767 (AFD-)	*ttx-1(p767) V*		M9 control	30 animals, each tested thrice	0.08889	0.02940		
44	JN1715 (AWB-)	*peIs1715*		M9 control	30 animals, each tested thrice	0.05556	0.02940		
45	CX4 (AWA-)	*odr-7(ky4) X*		M9 control	30 animals, each tested thrice	0.08889	0.04006		
46	PR672 (ASE-)	*che-1(p672) I*		M9 control	30 animals, each tested thrice	0.11111	0.04006		
47	N2			Predator cue	30 animals, each tested thrice	0.91111	0.02940		Fig. 2c

48	PY7502 (AWC-)	*oyIs85*		Predator cue	30 animals, each tested thrice	0.84444	0.02940		Fig. 2c
49	PR767 (AFD-)	*ttx-1(p767) V*		Predator cue	30 animals, each tested thrice	0.95556	0.02940		Fig. 2c
50	JN1715 (AWB-)	*peIs1715*		Predator cue	30 animals, each tested thrice	0.85556	0.02940		Fig. 2c
51	CX4 (AWA-)	*odr-7(ky4) X*		Predator cue	30 animals, each tested thrice	0.88889	0.04006		Fig. 2c
52	PR672 (ASE-)	*che-1(p672) I*		Predator cue	30 animals, each tested thrice	0.75556	0.04843		Fig. 2c
53	PR671	*tax-2 (p671)*		M9 control	30 animals, each tested thrice	0.04444	0.01111		
54	PR678	*tax-4 (p678)*		M9 control	30 animals, each tested thrice	0.07778	0.01111		
55	FK103	*tax-4 (ks28)*		M9 control	30 animals, each tested thrice	0.03333	0.01925		
56	CX6742	all *tax-4* neurons rescue		M9 control	30 animals, each tested thrice	0.13333	0.03849		
57	IV308	ASI rescue 100 ng/μl of *tax-4 (ks28)*		M9 control	30 animals, each tested thrice	0.04444	0.02940		
58	IV37	ASJ rescue 50ng/μl of *tax4 (ks28)*		M9 control	30 animals, each tested thrice	0.12222	0.02940		
59	IV78	ASH rescue 50ng/μl of *tax4 (ks28)*		M9 control	30 animals, each tested thrice	0.11111	0.02940		
60	N2			Predator cue	30 animals, each tested thrice	0.91111	0.04200		Fig. 3a, Supplementary Fig. S5a
61	PR671	*tax-2 (p671)*		Predator cue	30 animals, each tested thrice	0.66667	0.07070		Fig. 3a
62	PR678	*tax-4 (p678)*		Predator cue	30 animals, each tested thrice	0.17778	0.08000	significant compared to Exp#60	Fig. 3a
63	FK103	*tax-4 (ks28)*		Predator cue	30 animals, each tested thrice	0.11111	0.03510	significant compared to Exp#60	Fig. 3a, Supplementary Fig. S5a

64	CX6742	all *tax-4* neurons rescue		Predator cue	30 animals, each tested thrice	0.86667	0.04700	significant compared to Exp#63	Fig. 3a
65	IV698	ASI rescue 10ng/µl of *tax-4 (ks28)*		Predator cue	30 animals, each tested thrice	0.41111	0.06759	significant compared to Exp#60; significant compared to Exp#63	Supplementary Fig. S5a
66	IV700	ASI rescue 50ng/µl of *tax-4 (ks28)*		Predator cue	30 animals, each tested thrice	0.68889	0.05556	significant compared to Exp#60; significant compared to Exp#63	Supplementary Fig. S5a
67	IV308	ASI rescue 100 ng/µl of *tax-4 (ks28)*		Predator cue	30 animals, each tested thrice	0.84444	0.04400	significant compared to Exp#63	Fig. 3a
68	IV756	ASJ rescue 10ng/µl of *tax4 (ks28)*		Predator cue	30 animals, each tested thrice	0.78889	0.06759	significant compared to Exp#63	Supplementary Fig. S5a
69	IV757	ASJ rescue 25ng/µl of *tax4 (ks28)*		Predator cue	30 animals, each tested thrice	0.94444	0.02940	significant compared to Exp#63	Supplementary Fig. S5a
70	IV37	ASJ rescue 50ng/µl of *tax4 (ks28)*		Predator cue	30 animals, each tested thrice	0.76660	0.05660	significant compared to Exp#63	Fig. 3a
71	IV78	ASH rescue 50ng/µl of *tax4 (ks28)*		Predator cue	30 animals, each tested thrice	0.15556	0.04400	significant compared to Exp#60	Fig. 3a
72	CX10	*osm-9 (ky10)*		M9 control	30 animals, each tested thrice	0.02222	0.02222		
73	CX4544	*ocr-2 (ak47)*		M9 control	30 animals, each tested thrice	0.12222	0.02940		
74	IV223	ASH rescue 50ng/µl of *ocr-2 (ak47)*		M9 control	30 animals, each tested thrice	0.12222	0.02940		
75		ADL rescue 100ng/µl of		M9 control	30 animals, each tested thrice				

		ocr-2 (ak47)							
76	IV230	ASI rescue 50ng/µl of *ocr-2 (ak47)*		M9 control	30 animals, each tested thrice	0.01111	0.01111		
77	IV411	ASJ rescue 50ng/µl of *ocr-2 (ak47)*		M9 control	30 animals, each tested thrice	0.10000	0.05092		
78	N2			Predator cue	30 animals, each tested thrice	0.90000	0.03700		Fig. 3b, Supplementary Fig. S5b
79	CX10	*osm-9 (ky10)*		Predator cue	30 animals, each tested thrice	0.84444	0.05500		Fig. 3b, Supplementary Fig. S5b
80	CX4544	*ocr-2 (ak47)*		Predator cue	30 animals, each tested thrice	0.15556	0.05556	significant compared to Exp#78	Fig. 3b, Supplementary Fig. S5b
81	IV754	ASH rescue 10ng/µl of *ocr-2 (ak47)*		Predator cue	30 animals, each tested thrice	0.51111	0.04843	significant compared to Exp#78; significant compared to Exp#80	Supplementary Fig. S5b
82	IV755	ASH rescue 25ng/µl of *ocr-2 (ak47)*		Predator cue	30 animals, each tested thrice	0.71111	0.02940	significant compared to Exp#78; significant compared to Exp#80	Supplementary Fig. S5b
83	IV223	ASH rescue 50ng/µl of *ocr-2 (ak47)*		Predator cue	30 animals, each tested thrice	0.61111	0.06700	significant compared to Exp#78; significant compared to Exp#80	Fig. 3b
84	IV751	ADL rescue 10ng/µl of *ocr-2 (ak47)*		Predator cue	30 animals, each tested thrice	0.33333	0.06939	significant compared to Exp#78; significant compared to	Supplementary Fig. S5b

								Exp#80	
85	IV752	ADL rescue 25ng/µl of *ocr-2 (ak47)*		Predator cue	30 animals, each tested thrice	0.46667	0.05092	significant compared to Exp#78; significant compared to Exp#80	Supplementary Fig. S5b
86	IV753	ADL rescue 50ng/µl of *ocr-2 (ak47)*		Predator cue	30 animals, each tested thrice	0.38889	0.04843	significant compared to Exp#78; significant compared to Exp#80	Supplementary Fig. S5b
87	IV703	ADL rescue 100ng/µl of *ocr-2 (ak47)*		Predator cue	30 animals, each tested thrice	0.77778	0.04843	significant compared to Exp#80	Fig. 3b
88	IV230	ASI rescue 50ng/µl of *ocr-2 (ak47)*		Predator cue	30 animals, each tested thrice	0.06667	0.02300	significant compared to Exp#78	Fig. 3b
89	IV411	ASJ rescue 50ng/µl of *ocr-2 (ak47)*		Predator cue	30 animals, each tested thrice	0.07778	0.02200	significant compared to Exp#78	Fig. 3b
90	CX7154	*tax4 (ks28), ocr2 (ak47),*		M9 control	30 animals, each tested thrice	0.04444	0.02940		
91	IV260	*tax4 (ks28), ocr2 (ak47),* ASI rescue of *tax-4*		M9 control	30 animals, each tested thrice	0.12222	0.02222		
92	IV410	*tax4 (ks28), ocr2 (ak47),* ASJ rescue of *tax-4*		M9 control	30 animals, each tested thrice	0.07778	0.02940		
93	IV231	*tax4 (ks28), ocr2 (ak47),* ASH rescue of *ocr-2*		M9 control	30 animals, each tested thrice	0.16667	0.05092		
94	IV722	tax4/ocr2 (ks28/ak47), ADL rescue		M9 control	30 animals, each tested thrice	0.15556	0.02940		

		of ocr-2							
95	IV262	*tax4 (ks28), ocr2 (ak47),* ASI rescue of *tax-4*;ASH rescue of *ocr-2*		M9 control	30 animals, each tested thrice	0.08889	0.04843		
96	IV408	*tax4 (ks28), ocr2 (ak47),* ASJ rescue of *tax-4*;ASH rescue of *ocr-2*		M9 control	30 animals, each tested thrice	0.02222	0.01111		
97	IV719	*tax4 (ks28), ocr2 (ak47),* ASI rescue of *tax-4*;ADL rescue of *ocr-2*		M9 control	30 animals, each tested thrice	0.12222	0.02940		
98	IV721	*tax4 (ks28), ocr2 (ak47),* ASJ rescue of *tax-4*;ADL rescue of *ocr-2*		M9 control	30 animals, each tested thrice	0.13333	0.05092		
99	N2			Predator cue	30 animals, each tested thrice	0.94000	0.03700		Fig. 3c
100	CX7154	*tax4 (ks28), ocr2 (ak47)*		Predator cue	30 animals, each tested thrice	0.10000	0.05500	significant compared to Exp#99	Fig. 3c
101	IV260	*tax4 (ks28), ocr2 (ak47),* ASI rescue of *tax-4*		Predator cue	30 animals, each tested thrice	0.44000	0.07300	significant compared to Exp#99; significant compared to Exp#100	Fig. 3c
102	IV410	*tax4 (ks28), ocr2 (ak47),* ASJ rescue of		Predator cue	30 animals, each tested thrice	0.10000	0.04500	significant compared to Exp#99	Fig. 3c

		tax-4							
103	IV231	*tax4 (ks28), ocr2 (ak47),* ASH rescue of *ocr-2*		Predator cue	30 animals, each tested thrice	0.06600	0.02300	significant compared to Exp#99	Fig. 3c
104	IV722	*tax4 (ks28), ocr2 (ak47),* ADL rescue of *ocr-2*		Predator cue	30 animals, each tested thrice	0.18889	0.04843	significant compared to Exp#99	Fig. 3c
105	IV262	*tax4 (ks28), ocr2 (ak47),* ASI rescue of *tax-4*;ASH rescue of *ocr-2*		Predator cue	30 animals, each tested thrice	0.84000	0.06700	significant compared to Exp#100	Fig. 3c
106	IV408	*tax4 (ks28), ocr2 (ak47),* ASJ rescue of *tax-4*;ASH rescue of *ocr-2*		Predator cue	30 animals, each tested thrice	0.33000	0.07600	significant compared to Exp#99; significant compared to Exp#100	Fig. 3c
107	IV719	*tax4 (ks28), ocr2 (ak47),* ASI rescue of *tax-4*;ADL rescue of *ocr-2*		Predator cue	30 animals, each tested thrice	0.62222	0.06186	significant compared to Exp#99; significant compared to Exp#100	Fig. 3 c
108	IV721	*tax4 (ks28), ocr2 (ak47),* ASJ rescue of *tax-4;*ADL rescue of *ocr-2*		Predator cue	30 animals, each tested thrice	0.08889	0.02940	significant compared to Exp#99	Fig. 3c
109	N2		M9 control	Predator cue	100 animals, each tested thrice	0.88	0.031505		Fig. 4a
110	N2		1mM Sertraline	Predator cue	100 animals, each tested thrice	0.16333	0.037663	Significant compared to Exp#109	Fig. 4a
111	N2		M9 control	10mM $CuSO_4$	30 animals, each	1	0		Fig. 4a

					tested thrice				
112	N2		1mM Sertraline	10mM $CuSO_4$	30 animals, each tested thrice	0.677778	0.189541		Fig. 4a
113	N2		M9 control	1mM $CuSO_4$	30 animals, each tested thrice	0.8	0.088192		Fig. 4a
114	N2		1mM Sertraline	1mM $CuSO_4$	30 animals, each tested thrice	0.555556	0.164429		Fig. 4a
115	N2		M9 control	0.1mM $CuSO_4$	30 animals, each tested thrice	0.244444	0.221944		Fig. 4a
116	N2		1mM Sertraline	0.1mM $CuSO_4$	30 animals, each tested thrice	0.33333	0.202759		Fig. 4a
117	N2		M9 control	0.5M fructose	40 animals, each tested thrice	0.966667	0.013608		Fig. 4a
118	N2		1mM Sertraline	0.5M fructose	40 animals, each tested thrice	0.416667	0.051819	Significant compared to Exp#117	Fig. 4a
119	N2		M9 control	0.25M fructose	40 animals, tested thrice	0.816667	0.028868		Fig. 4a
120	N2		1mM Sertraline	0.25M fructose	40 animals, each tested thrice	0.316667	0.143049	Significant compared to Exp#119	Fig. 4a
121	N2		M9 control	0.125M fructose	30 animals, each tested thrice	0.555556	0.077778		Fig. 4a
122	N2		1mM Sertraline	0.125M fructose	30 animals, each tested thrice	0.244444	0.112765		Fig. 4a
123	FK103	*tax-4 (ks28)*	1mM Sertraline	Predator cue	30 animals, each tested thrice	0.18889	0.02940		Fig. 4b
124	IV37	ASJ rescue of *tax4 (ks28)*	M9 control	Predator cue	30 animals, each tested thrice	0.81111	0.04006		Fig. 4b
125	IV37	ASJ rescue of *tax4 (ks28)*	1mM Sertraline	Predator cue	30 animals, each tested thrice	0.31111	0.04843	significant compared to Exp#124	Fig. 4b
126	IV308	ASI rescue of *tax4 (ks28)*	M9 control	Predator cue	30 animals, each tested thrice	0.81111	0.02940		Fig. 4b
127	IV308	ASI rescue of *tax4 (ks28)*	1mM Sertraline	Predator cue	30 animals, each tested thrice	0.27778	0.02940	significant compared to Exp#126	Fig. 4b
128	CX4544	*ocr-2 (ak47)*	M9 control	Predator cue	30 animals, each	0.18889	0.07286		Fig. 4b

					tested thrice				
129	CX4544	*ocr-2 (ak47)*	1mM Sertraline	Predator cue	30 animals, each tested thrice	0.16667	0.01925		Fig. 4b
130	IV223	ASH rescue of *ocr-2 (ak47)*	M9 control	Predator cue	30 animals, each tested thrice	0.57778	0.04843		Fig. 4b
131	IV223	ASH rescue of *ocr-2 (ak47)*	1mM Sertraline	Predator cue	30 animals, each tested thrice	0.15556	0.02940	significant compared to Exp#130	Fig. 4b
132	IV703	ADL rescue of *ocr-2 (ak47)*	M9 control	Predator cue	30 animals, each tested thrice	0.74444	0.07778		Fig. 4b
133	IV703	ADL rescue of *ocr-2 (ak47)*	1mM Sertraline	Predator cue	30 animals, each tested thrice	0.35556	0.04006	significant compared to Exp#132	Fig. 4b
134	CX5	*eat-4 (ky5)*	M9 control	Predator cue	30 animals, each tested thrice	0.44444	0.04444		Fig. 4c
135	CX5	*eat-4 (ky5)*	1mM Sertraline	Predator cue	30 animals, each tested thrice	0.08889	0.04006	significant compared to Exp#134	Fig. 4c
136	MT5969	*unc-25 (n2328)*	M9 control	Predator cue	30 animals, each tested thrice	0.78889	0.07286		Fig. 4c
137	MT5969	*unc-25 (n2328)*	1mM Sertraline	Predator cue	30 animals, each tested thrice	0.72222	0.07286		Fig. 4c
138	MT5969	*unc-25 (n2328)*	pretreat with GABA 100mM, M9 control	Predator cue	30 animals, each tested thrice	0.77778	0.02940		Fig. 4c
139	MT5969	*unc-25 (n2328)*	pretreat with GABA 100mM, 1mM Sertraline	Predator cue	30 animals, each tested thrice	0.54444	0.07778		Fig. 4c
140	MT5969	*unc-25 (n2328)*	pretreat with GABA 300mM, M9 control	Predator cue	30 animals, each tested thrice	0.71111	0.06186		Fig. 4c
141	MT5969	*unc-25 (n2328)*	pretreat with GABA	Predator cue	30 animals, each tested thrice	0.17778	0.02940	significant compared to	Fig. 4c

			300mM, 1mM Sertraline					Exp#140	
142	IV625	*unc-25* rescue line1	M9 control	Predator cue	30 animals, each tested thrice	0.82222	0.04843		Fig. 4d
143	IV626	*unc-25* rescue line1	Sertraline	Predator cue	30 animals, each tested thrice	0.25556	0.04843	significant compared to Exp#142	Fig. 4d
144	IV625	*unc-25* rescue line2	M9 control	Predator cue	30 animals, each tested thrice	0.81111	0.02940		Fig. 4d
145	IV626	*unc-25* rescue line2	1mM Sertraline	Predator cue	30 animals, each tested thrice	0.21111	0.02940	significant compared to Exp#144	Fig. 4d
146	IV758	RIS rescue of *unc-25 (n2328)*	M9 control	Predator cue	30 animals, each tested thrice	0.76667	0.05092		Fig. 4d
147	IV758	RIS rescue of *unc-25 (n2328)*	1mM Sertraline	Predator cue	30 animals, each tested thrice	0.27778	0.04843	significant compared to Exp#146	Fig. 4d
148	IV787	RIS knockdown of *unc-25* line1	M9 control	Predator cue	60 animals, each tested thrice	0.894444	0.023439		Fig. 4d
149	IV787	RIS knockdown of *unc-25* line1	1mM sertraline	Predator cue	60 animals, each tested thrice	0.588889	0.09571	Significant compared to Exp#148	Fig. 4d
150	IV788	RIS knockdown of *unc-25* line2	M9 control	Predator cue	60 animals, each tested thrice	0.933333	0.014907		Fig. 4d
151	IV788	RIS knockdown of *unc-25* line2	1mM sertraline	Predator cue	60 animals, each tested thrice	0.611111	0.126978		Fig. 4d
152	BA1093	*snf-10 (hc194)*	M9 control	Predator cue	30 animals, each tested thrice	0.955556	0.011111		Fig. 4e
153	BA1093	*snf-10 (hc194)*	1mM sertraline	Predator cue	30 animals, each tested thrice	0.300000	0.029397	significant compared to Exp#152	Fig.4e
154	RM2710	*snf-11 (ok156)*	M9 control	Predator cue	30 animals, each tested thrice	0.88889	0.02940		Fig.4e
155	RM2710	*snf-11 (ok156)*	1mM sertraline	Predator cue	30 animals, each tested thrice	0.13333	0.05092	significant compared to	Fig.4e

								Exp#154	
156	CB177	*unc-46 (e177)*	M9 control	Predator cue	30 animals, each tested thrice	0.54444	0.02940		Fig.4e
157	CB177	*unc-46 (e177)*	1mM Sertraline	Predator cue	30 animals, each tested thrice	0.11111	0.02940	significant compared to Exp#156	Fig.4e
158	CB307	*unc-47 (e307)*	M9 control	Predator cue	30 animals, each tested thrice	0.70000	0.10715		Fig.4e
159	CB307	*unc-47 (e307)*	1mM Sertraline	Predator cue	30 animals, each tested thrice	0.12222	0.04006	significant compared to Exp#158	Fig.4e
160	N2			*P. pacificus* RS5275B secretions after 7hr on food	30 animals, each tested thrice	0.04444	0.02940		Supplementary Fig. S1a
161	N2			*P. pacificus* RS5275B secretions after 7hr off food	30 animals, each tested thrice	0.46667	0.06939		Supplementary Fig. S1a
162	N2			*P. pacificus* RS5275B secretions after 14hr on food	30 animals, each tested thrice	0.07778	0.01111		Supplementary Fig. S1a
163	N2			*P. pacificus* RS5275B secretions after 14hr off food	30 animals, each tested thrice	0.62222	0.06759		Supplementary Fig. S1a
164	N2			*P. pacificus* RS5275B secretions after 21hr on food	30 animals, each tested thrice	0.08889	0.02940		Supplementary Fig. S1a
165	N2			*P. pacificus* RS5275B secretions after 21hr off food	30 animals, each tested thrice	0.90000	0.03849		Supplementary Fig. S1a
166	N2			solvent control EtOH 1:7	30 animals, each tested thrice	0.17778	0.02940		
167	N2			solvent control EtOH 1:14	30 animals, each tested thrice	0.14444	0.02222		
168	N2			solvent control EtOH 1:28	30 animals, each tested thrice	0.10000	0.01925		
169	N2			solvent control EtOH 1:56	30 animals, each tested thrice	0.08889	0.01111		
170	N2			solvent control EtOH 1:112	30 animals, each tested thrice	0.11111	0.02940		

171	N2			solvent control EtOH 1:224	30 animals, each tested thrice	0.06667	0.01925		
172	N2			solvent control EtOH 1:448	30 animals, each tested thrice	0.08889	0.01111		
173	N2			solvent control EtOH 1:896	30 animals, each tested thrice	0.03333	0.01925		
174	N2			sulfate-containing compounds 1:7	30 animals, each tested thrice	1.00000	0.00000		Supplementary Fig. S3c
175	N2			sulfate-containing compound 1:14	30 animals, each tested thrice	0.98889	0.01111		Supplementary Fig. S3c
176	N2			sulfate-containing compound 1:28	30 animals, each tested thrice	0.96667	0.01925		Supplementary Fig. S3c
177	N2			sulfate-containing compound 1:56	30 animals, each tested thrice	0.97778	0.01111		Supplementary Fig. S3c
178	N2			sulfate-containing compound 1:112	30 animals, each tested thrice	0.94444	0.02940		Supplementary Fig. S3c
179	N2			sulfate-containing compound 1:224	30 animals, each tested thrice	0.88889	0.02940		Supplementary Fig. S3c
180	N2			sulfate-containing compound 1:448	30 animals, each tested thrice	0.83333	0.05092		Supplementary Fig. S3c
181	N2			sulfate-containing compound 1:896	30 animals, each tested thrice	0.35556	0.04006		Supplementary Fig. S3c
182	Mock			solvent control EtOH 1:112	35 animals, each tested thrice	0.03750	0.00686		
183	Mock			solvent control EtOH 1:448	28 animals, each tested thrice	0.03730	0.00913		
184	ASJ-			solvent control EtOH 1:112	10 animals, each tested thrice	0.02381	0.01065		
185	ASJ-			solvent control EtOH 1:448	11 animals, each tested thrice	0.06667	0.02843		
186	ASH-			solvent control EtOH 1:112	12 animals, each tested thrice	0.00000	0.00000		
187	ASH-			solvent control EtOH 1:448	11 animals, each tested thrice	0.06111	0.00237		
188	ASI-			solvent control EtOH 1:112	11 animals, each tested thrice	0.06667	0.02843		
189	ASI-			solvent control EtOH 1:448	10 animals, each tested thrice	0.06667	0.00000		
190	ADL-			solvent control EtOH	14 animals, each	0.07639	0.01312		

				1:112	tested thrice				
191	ADL-			solvent control EtOH 1:448	12 animals, each tested thrice	0.05556	0.02268		
192	ADF-			solvent control EtOH 1:112	11 animals, each tested thrice	0.06111	0.00237		
193	ADF-			solvent control EtOH 1:448	11 animals, each tested thrice	0.06548	0.00761		
194	ASG-			solvent control EtOH 1:112	9 animals, each tested thrice	0.15000	0.00786		
195	ASG-			solvent control EtOH 1:448	9 animals, each tested thrice	0.08333	0.01309		
196	ASK-			solvent control EtOH 1:112	9 animals, each tested thrice	0.07500	0.00393		
197	ASK-			solvent control EtOH 1:448	9 animals, each tested thrice	0.05556	0.02619		
198	Mock			sulfate-containing compound 1:112	35 animals, each tested thrice	0.89167	0.01969		Supplementary Fig. S3d
199	Mock			sulfate-containing compound 1:448	28 animals, each tested thrice	0.88095	0.02549		Supplementary Fig. S3d
200	ASJ-			sulfate-containing compound 1:112	10 animals, each tested thrice	0.46032	0.00710	significant compared to Exp#198	Supplementary Fig. S3d
201	ASJ-			sulfate-containing compound 1:448	11 animals, each tested thrice	0.48889	0.01895	significant compared to Exp#199	Supplementary Fig. S3d
202	ASH-			sulfate-containing compound 1:112	12 animals, each tested thrice	0.29048	0.03694	significant compared to Exp#198	Supplementary Fig. S3d
203	ASH-			sulfate-containing compound 1:448	11 animals, each tested thrice	0.34444	0.05212	significant compared to Exp#199	Supplementary Fig. S3d
204	ASI-			sulfate-containing compound 1:112	11 animals, each tested thrice	0.58333	0.03553	significant compared to Exp#198	Supplementary Fig. S3d
205	ASI-			sulfate-containing compound 1:448	10 animals, each tested thrice	0.76667	0.04472	significant compared to Exp#199	Supplementary Fig. S3d
206	ADL-			sulfate-containing compound 1:112	14 animals, each tested thrice	0.31250	0.07087	significant compared to	Supplementary Fig. S3d

								Exp#198	
207	ADL-			sulfate-containing compound 1:448	12 animals, each tested thrice	0.72222	0.00000		Supplementary Fig. S3d
208	ADF-			sulfate-containing compound 1:112	11 animals, each tested thrice	0.91111	0.00948		Supplementary Fig. S3d
209	ADF-			sulfate-containing compound 1:448	11 animals, each tested thrice		0.03046		Supplementary Fig. S3d
210	ASG-			sulfate-containing compound 1:112	9 animals, each tested thrice	0.88333	0.02357		Supplementary Fig. S3d
212	ASG-			sulfate-containing compound 1:448	9 animals, each tested thrice	0.94444	0.02619		Supplementary Fig. S3d
212	ASK-			sulfate-containing compound 1:112	9 animals, each tested thrice	0.95833	0.01964		Supplementary Fig. S3d
213	ASK-			sulfate-containing compound 1:448	9 animals, each tested thrice	1.00000	0.00000		Supplementary Fig. S3d
214	N2			solvent control EtOH 1:112	30 animals, each tested thrice	0.11111	0.01111		
215	N2			solvent control EtOH 1:448	30 animals, each tested thrice	0.03333	0.01925		
216	PY7502 (AWC-)	*oyIs85*		solvent control EtOH 1:112	30 animals, each tested thrice	0.07778	0.01111		
217	PY7502 (AWC-)	*oyIs85*		solvent control EtOH 1:448	30 animals, each tested thrice	0.06667	0.01925		
218	PR767 (AFD-)	*ttx-1(p767) V*		solvent control EtOH 1:112	30 animals, each tested thrice	0.08889	0.01111		
219	PR767 (AFD-)	*ttx-1(p767) V*		solvent control EtOH 1:448	30 animals, each tested thrice	0.10000	0.01925		
220	JN1715 (AWB-)	*peIs1715*		solvent control EtOH 1:112	30 animals, each tested thrice	0.08889	0.02222		
221	JN1715 (AWB-)	*peIs1715*		solvent control EtOH 1:448	30 animals, each tested thrice	0.04444	0.01111		
222	CX4 (AWA-)	*odr-7(ky4) X*		solvent control EtOH 1:112	30 animals, each tested thrice	0.12222	0.02222		
223	CX4 (AWA-)	*odr-7(ky4) X*		solvent control EtOH 1:448	30 animals, each tested thrice	0.03333	0.01925		
224	PR672 (ASE-)	*che-1(p672) I*		solvent control EtOH 1:112	30 animals, each tested thrice	0.05556	0.01111		
225	PR672 (ASE-)	*che-1(p672) I*		solvent control EtOH 1:448	30 animals, each tested thrice	0.02222	0.01111		

226	N2			sulfate-containing compound 1:112	30 animals, each tested thrice	0.97778	0.01111		Supplementary Fig. S3e
227	N2			sulfate-containing compound 1:448	30 animals, each tested thrice	0.86667	0.05774		Supplementary Fig. S3e
228	PY7502 (AWC-)	*oyIs85*		sulfate-containing compound 1:112	30 animals, each tested thrice	0.96667	0.01925		Supplementary Fig. S3e
229	PY7502 (AWC-)	*oyIs85*		sulfate-containing compound 1:448	30 animals, each tested thrice	0.87778	0.04843		Supplementary Fig. S3e
230	PR767 (AFD-)	*ttx-1(p767) V*		sulfate-containing compound 1:112	30 animals, each tested thrice	0.98889	0.01111		Supplementary Fig. S3e
231	PR767 (AFD-)	*ttx-1(p767) V*		sulfate-containing compound 1:448	30 animals, each tested thrice	0.94444	0.02940		Supplementary Fig. S3e
232	JN1715 (AWB-)	*peIs1715*		sulfate-containing compound 1:112	30 animals, each tested thrice	0.87778	0.02940		Supplementary Fig. S3e
233	JN1715 (AWB-)	*peIs1715*		sulfate-containing compound 1:448	30 animals, each tested thrice	0.65556	0.08678		Supplementary Fig. S3e
234	CX4 (AWA-)	*odr-7(ky4) X*		sulfate-containing compound 1:112	30 animals, each tested thrice	0.97778	0.02222		Supplementary Fig. S3e
235	CX4 (AWA-)	*odr-7(ky4) X*		sulfate-containing compound 1:448	30 animals, each tested thrice	0.80000	0.08389		Supplementary Fig. S3e
236	PR672 (ASE-)	*che-1(p672) I*		sulfate-containing compound 1:112	30 animals, each tested thrice	0.67778	0.05879	significant compared to Exp#226	Supplementary Fig. S3e
237	PR672 (ASE-)	*che-1(p672) I*		sulfate-containing compound 1:448	30 animals, each tested thrice	0.35556	0.05879	significant compared to Exp#227	Supplementary Fig. S3e
238	Mock			M9 control	59 animals, each tested thrice	0.04722	0.01389		
239	ASJ-			M9 control	19 animals, each tested thrice	0.10000	0.06667		
240	ASH-			M9 control	19 animals, each tested thrice	0.02083	0.02083		
241	ASI-			M9 control	22 animals, each tested thrice	0.06111	0.00556		
242	ADL-			M9 control	21 animals, each tested thrice	0.07371	0.01657		
243	ADF-			M9 control	21 animals, each tested thrice	0.08194	0.02639		
244	ASG-			M9 control	20 animals, each	0.12738	0.05595		

					tested thrice				
245	ASK-			M9 control	11 animals, each tested thrice	0.15556	0.04444		
246	Mock			1.0 mM SDS	32 animals, each tested thrice	0.87619	0.02974		Supplementary Fig. S3f
247	Mock			0.1 mM SDS	27 animals, each tested thrice	0.68333	0.07097		Supplementary Fig. S3f
248	ASJ-			1.0 mM SDS	10 animals, each tested thrice	0.53333	0.00000		Supplementary Fig. S3f
249	ASJ-			0.1 mM SDS	9 animals, each tested thrice	0.25000	0.08333	significant compared to Exp#247	Supplementary Fig. S3f
250	ASH-			1.0 mM SDS	9 animals, each tested thrice	0.48333	0.01667	significant compared to Exp#246	Supplementary Fig. S3f
251	ASH-			0.1 mM SDS	10 animals, each tested thrice	0.06944	0.01389	significant compared to Exp#247	Supplementary Fig. S3f
252	ASI-			1.0 mM SDS	12 animals, each tested thrice	0.87619	0.07619		Supplementary Fig. S3f
253	ASI-			0.1 mM SDS	10 animals, each tested thrice	0.31944	0.06944	significant compared to Exp#247	Supplementary Fig. S3f
254	ADL-			1.0 mM SDS	12 animals, each tested thrice	0.76667	0.10000		Supplementary Fig. S3f
255	ADL-			0.1 mM SDS	9 animals, each tested thrice	0.68056	0.06944		Supplementary Fig. S3f
256	ADF-			1.0 mM SDS	12 animals, each tested thrice	0.83333	0.05556		Supplementary Fig. S3f
257	ADF-			0.1 mM SDS	9 animals, each tested thrice	0.62500	0.04167		Supplementary Fig. S3f
258	ASG-			1.0 mM SDS	11 animals, each tested thrice	0.95238	0.04762		Supplementary Fig. S3f
259	ASG-			0.1 mM SDS	9 animals, each tested thrice	0.81667	0.01667		Supplementary Fig. S3f
260	ASK-			1.0 mM SDS	10 animals, each tested thrice	0.83333	0.00000		Supplementary Fig. S3f
261	ASK-			0.1 mM SDS	11 animals, each tested thrice	0.83333	0.05000		Supplementary Fig. S3f

262	N2			M9 control	30 animals, each tested thrice	0.08889	0.01111		
263	PY7502 (AWC-)	*oyIs85*		M9 control	30 animals, each tested thrice	0.11111	0.02940		
264	PR767 (AFD-)	*ttx-1(p767) V*		M9 control	30 animals, each tested thrice	0.08889	0.02940		
265	JN1715 (AWB-)	*peIs1715*		M9 control	30 animals, each tested thrice	0.05556	0.02940		
266	CX4 (AWA-)	*odr-7(ky4) X*		M9 control	30 animals, each tested thrice	0.08889	0.04006		
267	PR672 (ASE-)	*che-1(p672) I*		M9 control	30 animals, each tested thrice	0.11111	0.04006		
268	N2			1.0 mM SDS	30 animals, each tested thrice	0.92222	0.02222		Supplementary Fig. S3g
269	N2			0.1 mM SDS	30 animals, each tested thrice	0.38889	0.04843		Supplementary Fig. S3g
270	PY7502 (AWC-)	*oyIs85*		1.0 mM SDS	30 animals, each tested thrice	0.85556	0.01111		Supplementary Fig. S3g
271	PY7502 (AWC-)	*oyIs85*		0.1 mM SDS	30 animals, each tested thrice	0.93333	0.05092	significant compared to Exp#269	Supplementary Fig. S3g
272	PR767 (AFD-)	*ttx-1(p767) V*		1.0 mM SDS	30 animals, each tested thrice	0.92222	0.04006		Supplementary Fig. S3g
273	PR767 (AFD-)	*ttx-1(p767) V*		0.1 mM SDS	30 animals, each tested thrice	0.28889	0.07286		Supplementary Fig. S3g
274	JN1715 (AWB-)	*peIs1715*		1.0 mM SDS	30 animals, each tested thrice	0.90000	0.05092		Supplementary Fig. S3g
275	JN1715 (AWB-)	*peIs1715*		0.1 mM SDS	30 animals, each tested thrice	0.44444	0.19277		Supplementary Fig. S3g
276	CX4 (AWA-)	*odr-7(ky4) X*		1.0 mM SDS	30 animals, each tested thrice	0.88889	0.02940		Supplementary Fig. S3g
277	CX4 (AWA-)	*odr-7(ky4) X*		0.1 mM SDS	30 animals, each tested thrice	0.25556	0.04843		Supplementary Fig. S3g
278	PR672 (ASE-)	*che-1(p672) I*		1.0 mM SDS	30 animals, each tested thrice	0.86667	0.05774		Supplementary Fig. S3g
279	PR672 (ASE-)	*che-1(p672) I*		0.1 mM SDS	30 animals, each tested thrice	0.20000	0.03849		Supplementary Fig. S3g

280	N2			solvent control EtOH 1:112	30 animals, each tested thrice	0.11111	0.01111		
281	N2			solvent control EtOH 1:448	30 animals, each tested thrice	0.03333	0.01925		
282	PR671	*tax-2 (p671)*		solvent control EtOH 1:112	30 animals, each tested thrice	0.06667	0.01925		
283	PR671	*tax-2 (p671)*		solvent control EtOH 1:448	30 animals, each tested thrice	0.04444	0.01111		
284	FK103	*tax-4 (ks28)*		solvent control EtOH 1:112	30 animals, each tested thrice	0.03333	0.01925		
285	FK103	*tax-4 (ks28)*		solvent control EtOH 1:448	30 animals, each tested thrice	0.05556	0.02222		
286	CX10	*osm-9 (ky10)*		solvent control EtOH 1:112	30 animals, each tested thrice	0.07778	0.02940		
287	CX10	*osm-9 (ky10)*		solvent control EtOH 1:448	30 animals, each tested thrice	0.04444	0.01111		
288	CX4544	*ocr-2 (ak47)*		solvent control EtOH 1:112	30 animals, each tested thrice	0.08889	0.04006		
289	CX4544	*ocr-2 (ak47)*		solvent control EtOH 1:448	30 animals, each tested thrice	0.07778	0.02940		
290	N2			sulfate-containing compound 1:112	30 animals, each tested thrice	0.97778	0.01111		Supplementary Fig. S5c
291	N2			sulfate-containing compound 1:448	30 animals, each tested thrice	0.82222	0.04006		Supplementary Fig. S5c
292	PR671	*tax-2 (p671)*		sulfate-containing compound 1:112	30 animals, each tested thrice	0.94444	0.02940		Supplementary Fig. S5c
293	PR671	*tax-2 (p671)*		sulfate-containing compound 1:448	30 animals, each tested thrice	0.77778	0.04006		Supplementary Fig. S5c
294	FK103	*tax-4 (ks28)*		sulfate-containing compound 1:112	30 animals, each tested thrice	0.32222	0.04006	significant compared to Exp#290	Supplementary Fig. S5c
295	FK103	*tax-4 (ks28)*		sulfate-containing compound 1:448	30 animals, each tested thrice	0.08889	0.01111	significant compared to Exp#291	Supplementary Fig. S5c
296	CX10	*osm-9 (ky10)*		sulfate-containing compound 1:112	30 animals, each tested thrice	0.64444	0.04843	significant compared to Exp#290	Supplementary Fig. S5c
297	CX10	*osm-9 (ky10)*		sulfate-containing compound 1:448	30 animals, each tested thrice	0.40000	0.05092	significant compared to	Supplementary Fig. S5c

								Exp#291	
298	CX4544	*ocr-2 (ak47)*		sulfate-containing compound 1:112	30 animals, each tested thrice	0.22222	0.04006	significant compared to Exp#290	Supplementary Fig. S5c
299	CX4544	ocr-2 (ak47)		sulfate-containing compound 1:448	30 animals, each tested thrice	0.10000	0.01925	significant compared to Exp#291	Supplementary Fig. S5c
300	N2		M9 control	Predator cue	30 animals, each tested thrice	0.88889	0.04006		Supplementary Fig. S6c
301	N2		1mM Sertraline	Predator cue	30 animals, each tested thrice	0.18889	0.04006	significant compared to Exp#300	Supplementary Fig. S6c
302	N2		M9 control, test after 15min	Predator cue	30 animals, each tested thrice	0.90000	0.05092		Supplementary Fig. S6c
303	N2		Sertraline 1mM, test after 15min	Predator cue	30 animals, each tested thrice	0.53333	0.05092	significant compared to Exp#302	Supplementary Fig. S6c
304	N2		M9 control, test after 30min	Predator cue	30 animals, each tested thrice	0.92222	0.04006		Supplementary Fig. S6c
305	N2		Sertraline 1mM, test after 30min	Predator cue	30 animals, each tested thrice	0.67778	0.04843	significant compared to Exp#304	Supplementary Fig. S6c
306	N2		M9 control, test after 45min	Predator cue	30 animals, each tested thrice	0.92222	0.04006		Supplementary Fig. S6c
307	N2		Sertraline 1mM, test after 45min	Predator cue	30 animals, each tested thrice	0.86667	0.05092		Supplementary Fig. S6c
308	N2		M9 control	sulfate-containing compound 1:112	30 animals, each tested thrice	0.97778	0.01111		Supplementary Fig. S6a
309	N2		M9 control	sulfate-containing compound 1:448	30 animals, each tested thrice	0.88889	0.02940		Supplementary Fig. S6a
310	N2		1mM Sertraline	sulfate-containing compound 1:112	30 animals, each tested thrice	0.15556	0.02940	significant compared to Exp#308	Supplementary Fig. S6a
311	N2		1mM Sertraline	sulfate-containing compound 1:448	30 animals, each tested thrice	0.12222	0.04006	significant compared to	Supplementary Fig. S6a

								Exp#309	
312	N2		M9 control	Predator cue	30 animals, each tested thrice	0.91111	0.04006		Supplementary Fig. S6b
313	N2		Sertraline 0.25mM	Predator cue	30 animals, each tested thrice		0.02940		Supplementary Fig. S6b
314	N2		Sertraline 0.5mM	Predator cue	30 animals, each tested thrice	0.71111	0.02940	significant compared to Exp#312	Supplementary Fig. S6b
315	N2		Sertraline 1mM	Predator cue	30 animals, each tested thrice	0.14444	0.02940	significant compared to Exp#312	Supplementary Fig. S6b
316	N2		Sertraline 2mM	Predator cue	30 animals, each tested thrice	0.12222	0.02940	significant compared to Exp#312	Supplementary Fig. S6b
317	N2		M9 control	Predator cue	30 animals, each tested thrice	0.84444	0.04006		Supplementary Fig. S6d
318	N2		1mM Sertraline	Predator cue	30 animals, each tested thrice	0.17778	0.05879	significant compared to Exp#317	Supplementary Fig. S6d
319	GR1321	tph-1 (mg280)	M9 control	Predator cue	30 animals, each tested thrice	0.72222	0.06759		Supplementary Fig. S6d
320	GR1321	tph-1 (mg280)	1mM Sertraline	Predator cue	30 animals, each tested thrice	0.17778	0.05879	significant compared to Exp#319	Supplementary Fig. S6d
321	MT9772	mod-5 (n3314)	M9 control	Predator cue	30 animals, each tested thrice	0.84444	0.02940		Supplementary Fig. S6d
322	MT9772	mod-5 (n3314)	1mM Sertraline	Predator cue	30 animals, each tested thrice	0.15556	0.02222	significant compared to Exp#331	Supplementary Fig. S6d
323	MT8944	mod-5 (n822)	M9 control	Predator cue	30 animals, each tested thrice	0.82222	0.02940		Supplementary Fig. S6d
324	MT8944	mod-5 (n822)	1mM Sertraline	Predator cue	30 animals, each tested thrice	0.17778	0.02940	significant compared to Exp#323	Supplementary Fig. S6d
325	MT13113	tdc-1 (n3419)	M9 control	Predator cue	30 animals, each tested thrice	0.84444	0.02940		Supplementary Fig. S6d
326	MT13113	tdc-1 (n3419)	1mM Sertraline	Predator cue	30 animals, each tested thrice	0.30000	0.07698	significant compared to Exp#325	Supplementary Fig. S6d

327	MT9455	tbh-1 (n3247)	M9 control	Predator cue	30 animals, each tested thrice	0.85556	0.04843		Supplementary Fig. S6d
328	MT9455	tbh-1 (n3247)	1mM Sertraline	Predator cue	30 animals, each tested thrice	0.30000	0.05092	significant compared to Exp#327	Supplementary Fig. S6d
329	CB1112	cat-2 (e1112)	M9 control	Predator cue	30 animals, each tested thrice	0.62222	0.05879		Supplementary Fig. S6d
330	CB1112	cat-2 (e1112)	1mM Sertraline	Predator cue	30 animals, each tested thrice	0.13333	0.01925	significant compared to Exp#329	Supplementary Fig. S6d
331	RM2702	dat-1 (ok157)	M9 control	Predator cue	30 animals, each tested thrice	0.91111	0.02940		Supplementary Fig. S6d
332	RM2702	dat-1 (ok157)	1mM Sertraline	Predator cue	30 animals, each tested thrice	0.32222	0.07778	significant compared to Exp#331	Supplementary Fig. S6d

Supplementary Table S8. Table showing all strains and their genotypes used in this study

Strain	Genotype	Location in the paper
N2	*C. elegans* isolate (England)	Secretions collected in Fig. 1a-b, Supplementary Fig. S1d, Animals tested in Figs. 1a-b, 2b-c, 3a-c, 3e, 4a-f, Supplementary Figs. S1a-d, S3b-g, S5a-c, S6a-d. "WT" or "Control".
PS312	*P. pacificus* wild isolate (California)	Secretions collected in Fig. 1a
RS5275B	*P. pacificus wild isolate (Bolivia)*	Secretions collected in Fig. 1a and termed "predator cue" in the rest of the figures.
PY7502	*oyIs85 [ceh-36p::TU#813 + ceh-36p::TU#814 + srtx-1p::GFP + unc-122p::dsRed]*	"AWC-", Fig. 2c, Supplementary Figs. S3e, S3g.
PR767	*ttx-1(p767) V*	"AFD-", Fig. 2c, Supplementary Figs. S3 e, S3g.
JN1715	*peIs1715 [str-1p::mCasp-1; unc-122p::venus]*	"AWB-", Fig. 2c, Supplementary Figs. S3 e, S3g.
CX4	*odr-7(ky4) X*	"AWA-", Fig. 2c, Supplementary Figs. S3 e, S3g.
PR672	*che-1(p672) I*	"ASE-", Fig. 2c, Supplementary Figs. S3 e, S3g.
CX12022	*kyEx3290[sre-1::GCaMP3; unc-122::dsRed2]*	Fig. 2d, Supplementary Fig. S4a.
IV346	*kyEx2865 [sra-6::gcamp3; unc-122::gfp]*	Figs. 2e, 2f, Supplementary Figs. S4b-c.
ZD1184	*qdEx103[trx-1p::GCaMP5;ofm-1::gfp]*	Fig. 2g, Supplementary Fig. S4d.
PR671	*tax-2 (p671) I*	"*tax-2*", Fig. 3a, Supplementary Fig. S5c.
PR678	*tax-4 (p678) III*	"*tax-4*", Fig. 3a.
FK103	*tax-4 (ks28) III*	"*tax-4*", Figs. 3a, 3e, 4b, Supplementary Figs. S5a, S5c.
CX6742	*tax-4 (ks28) III; kyEx744 [tax-4::tax-4::GFP, unc-122::gfp]*	"all *tax-4* neurons", Fig. 3a.
IV698	*tax-4(ks28) III; ueEx488 [str-3::tax-4::sl2gfp, elt-2::gfp]*	"ASI *tax-4* rescue (10ng/µl)", Supplementary Fig. S5a.
IV700	*tax-4(ks28) III; ueEx490 [str-3::tax-4::sl2gfp, elt-2::gfp]*	"ASI *tax-4* rescue (50ng/µl)", Supplementary Fig. S5a.
IV308	*tax-4(ks28) III; ueEx188 [str-3::tax-4::sl2gfp, elt-2::gfp]*	"ASI *tax-4* rescue (100ng/µl)", Figs. 3a, 3e, 4b.
IV756	*tax-4(ks28); ueEx545[srh-11p::tax-4::sl2gfp, elt-2::gfp]*	"ASJ *tax-4* rescue (10ng/µl)", Supplementary Fig. S5a.

IV757	*tax-4(ks28); ueEx546[srh-11p::tax-4::sl2gfp, elt-2::gfp]*	"ASJ *tax-4* rescue (25ng/µl)", Supplementary Fig. S5a.
IV37	*tax-4(ks28) III; ueEx17 [srh-11::tax-4::sl2gfp, elt-2::gfp]*	"ASJ *tax-4* rescue (50ng/µl)", Figs. 3a, 3e, 4b.
IV78	*tax-4(ks28) III; ueEx32 [sra-6::tax-4:sl2gfp, elt-2::gfp]*	"ASH *tax-4* rescue", Fig. 3a
CX10	*osm-9 (ky10) IV*	"*osm-9*", Fig. 3b, Supplementary Fig. S5c.
CX4544	*ocr-2 (ak47) IV*	"*ocr-2*", Figs. 3b, 3e, 4b, Supplementary Fig. S5b-c.
IV754	*ocr-2 (ak47) IV; ueEx543[sra-6p::ocr-2::sl2gfp, elt-2::gfp]*	"ASH *ocr-2* rescue (10ng/µl)", Supplementary Fig. S5b.
IV755	*ocr-2 (ak47) IV; ueEx544[sra-6p::ocr-2::sl2gfp, elt-2::gfp]*	"ASH *ocr-2* rescue (25ng/µl)", Supplementary Fig. S5b.
IV223	*ocr-2(ak47) IV; ueEx138 [sra-6::ocr-2::sl2gfp, elt-2::gfp]*	"ASH *ocr-2* rescue (50ng/µl)", Figs. 3b, 4b.
IV751	*ocr-2 (ak47) IV; ueEx540[sre-1p::ocr-2::sl2gfp, elt-2::gfp]*	"ADL *ocr-2* rescue (10ng/µl)", Supplementary Fig. S5b.
IV752	*ocr-2 (ak47) IV; ueEx541[sre-1p::ocr-2::sl2gfp, elt-2::gfp]*	"ADL *ocr-2* rescue (25ng/µl)", Supplementary Fig. S5b.
IV753	*ocr-2 (ak47) IV; ueEx542[sre-1p::ocr-2::sl2gfp, elt-2::gfp]*	"ADL *ocr-2* rescue (50ng/µl)", Supplementary Fig. S5b.
IV703	*ocr-2 (ak47) IV; ueEx493[sre-1p::ocr-2::sl2gfp, elt-2::gfp]*	"ADL *ocr-2* rescue (100ng/µl)", Figs. 3b, 4b.
IV230	*ocr-2(ak47) IV; ueEx145 [str-3::ocr-2::sl2gfp, elt-2::gfp]*	"ASI *ocr-2* rescue", Fig. 3b.
IV411	*ocr-2(ak47) IV; ueEx252 [srh-11::ocr-2::sl2gfp, elt-2::gfp]*	"ASJ *ocr-2* rescue", Fig. 3b.
CX7154	*tax-4(ks28) III; ocr-2(ak47) IV*	"*tax-4;ocr-2* double mutant", Fig. 3c.
IV722	*tax-4(ks28) III; ocr-2(ak47) IV; ueEx512[sre-1::ocr-2::sl2gfp, elt2gfp]*	"ADL *ocr-2* rescue in *tax-4;ocr-2* double mutant", Fig. 3c.
IV231	*tax-4(ks28) III; ocr-2(ak47) IV; ueEx146 [sra-6::ocr-2::sl2gfp, elt-2::gfp]*	"ASH *ocr-2* rescue in *tax-4;ocr-2* double mutant", Fig. 3c.
IV260	*tax-4(ks28) III; ocr-2(ak47) IV; ueEx166 [str-3::tax-4::sl2gfp, elt-2::gfp]*	"ASI *tax-4* rescue in *tax-4;ocr-2* double mutant", Fig. 3c.
IV410	*tax-4(ks28) III; ocr-2(ak47) IV; ueEx251 [srh-11::tax-4::sl2gfp, elt-2::gfp]*	"ASJ *tax-4* rescue in *tax-4;ocr-2* double mutant", Fig. 3c.
IV262	*tax-4(ks28) III; ocr-2(ak47) IV; ueEx168 [str-3::tax-4::sl2gfp, sra-6::ocr-2::sl2gfp, elt-2::gfp]*	"ASH *ocr-2* and ASI *tax-4* rescue in *tax-4;ocr-2* double mutant", Fig. 3c.
IV408	*tax-4(ks28) III; ocr-2(ak47) IV;*	"ASH *ocr-2* and ASJ *tax-4* rescue in *tax-*

	ueEx249 [srh-11::tax-4::sl2gfp; sra-6::ocr-2::sl2gfp, elt-2::gfp]	*4;ocr-2* double mutant", Fig. 3c.
IV719	*tax-4(ks28) III; ocr-2(ak47) IV; ueEx509[str-3::tax-4::sl2gfp, sre-1::ocr-2::sl2gfp, elt2gfp]*	"ADL *ocr-2* and ASI *tax-4* rescue in *tax-4;ocr-2* double mutant", Fig. 3c.
IV721	*tax-4(ks28) III; ocr-2(ak47) IV; ueEx511[srh-11::tax-4::sl2gfp, sre-1::ocr-2::sl2gfp, elt2gfp]*	"ADL *ocr-2* and ASJ *tax-4* rescue in *tax-4;ocr-2* double mutant", Fig. 3c.
CX5	*eat-4 (ky5) III*	"*eat-4*", Fig. 4c.
MT5969	*unc-25 (n2328) III*	"*unc-25*", Fig. 4c.
IV625	*unc-25 (n2328) III; ueEx418 [unc-25::unc-25cDNA, elt-2::gfp]*	"*unc-25p::unc-25(#1)*", Fig. 4d.
IV626	*unc-25 (n2328) III; ueEx419 [unc-25::unc-25cDNA, elt-2::gfp]*	"*unc-25p::unc-25(#2)*", Fig. 4d.
IV758	*unc-25(n2328); ueEx547[aptf-1p::unc-25::sl2gfp, elt-2::gfp]*	"*RIS::unc-25*", Fig. 4d.
IV787	*ueEx568[aptf-1p::unc25cDNA::sl2gfp]ueEx569[aptf-1p::unc-25antisense::sl2gfp; elt2::gfp]*	*"RIS::unc-25 KD #1"*, Fig 4d.
IV788	*ueEx568[aptf-1p::unc25cDNA::sl2gfp]ueEx569[aptf-1p::unc-25antisense::sl2gfp; elt2::gfp]*	*"RIS::unc-25 KD #2"*, Fig 4d.
CB177	*unc-46(e177) V*	"*unc-46*", Fig. 4e.
CB307	*unc-47(e307)III*	"*unc-47*", Fig. 4e.
RM2710	*snf-11(ok156) V*	"*snf-11*", Fig. 4e.
BA1093	*snf-10(hc194) V*	"*snf-10*", Fig. 4e.
CB4856	*C. elegans* wild isolate (Hawaii)	Supplementary Fig. S1b.
JU775	*C. elegans* wild isolate (Paris)	Supplementary Fig. S1b.
KR314	*C. elegans* wild isolate (Vancouver)	Supplementary Fig. S1b.
AB1	*C. elegans* wild isolate (Australia)	Supplementary Fig. S1b.
QX1211	*C. elegans* wild isolate (Princeton)	Supplementary Fig. S1b.
DL238	*C. elegans* wild isolate (Princeton)	Supplementary Fig. S1b.
CB4852	*C. elegans* wild isolate (England)	Supplementary Fig. S1b.
RC301	*C. elegans* wild isolate (Germany)	Supplementary Fig. S1b.
GR1321	*tph-1 (mg280) II*	"*tph-1*", Supplementary Fig. S6d.
MT9772	*mod-5 (n3314) I*	"*mod-5*", Supplementary Fig. S6d.
MT8944	*mod-5 (n822) I*	"*mod-5*", Supplementary Fig. S6d.
MT13113	*tdc-1 (n3419) II*	"*tdc-1*", Supplementary Fig. S6d.
MT9455	*tbh-1 (n3247) X*	"*tbh-1*", Supplementary Fig. S6d.
CB1112	*cat-2 (e1112) II*	"*cat-2*", Supplementary Fig. S6d.
RM2702	*dat-1 (ok157) III*	"*dat-1*", Supplementary Fig. S6d.

3. Supplementary Methods

^{1}H NMR spectrum of active metabolome fraction containing sufac#1 and sufal#2 (600 MHz, methanol-d_4).

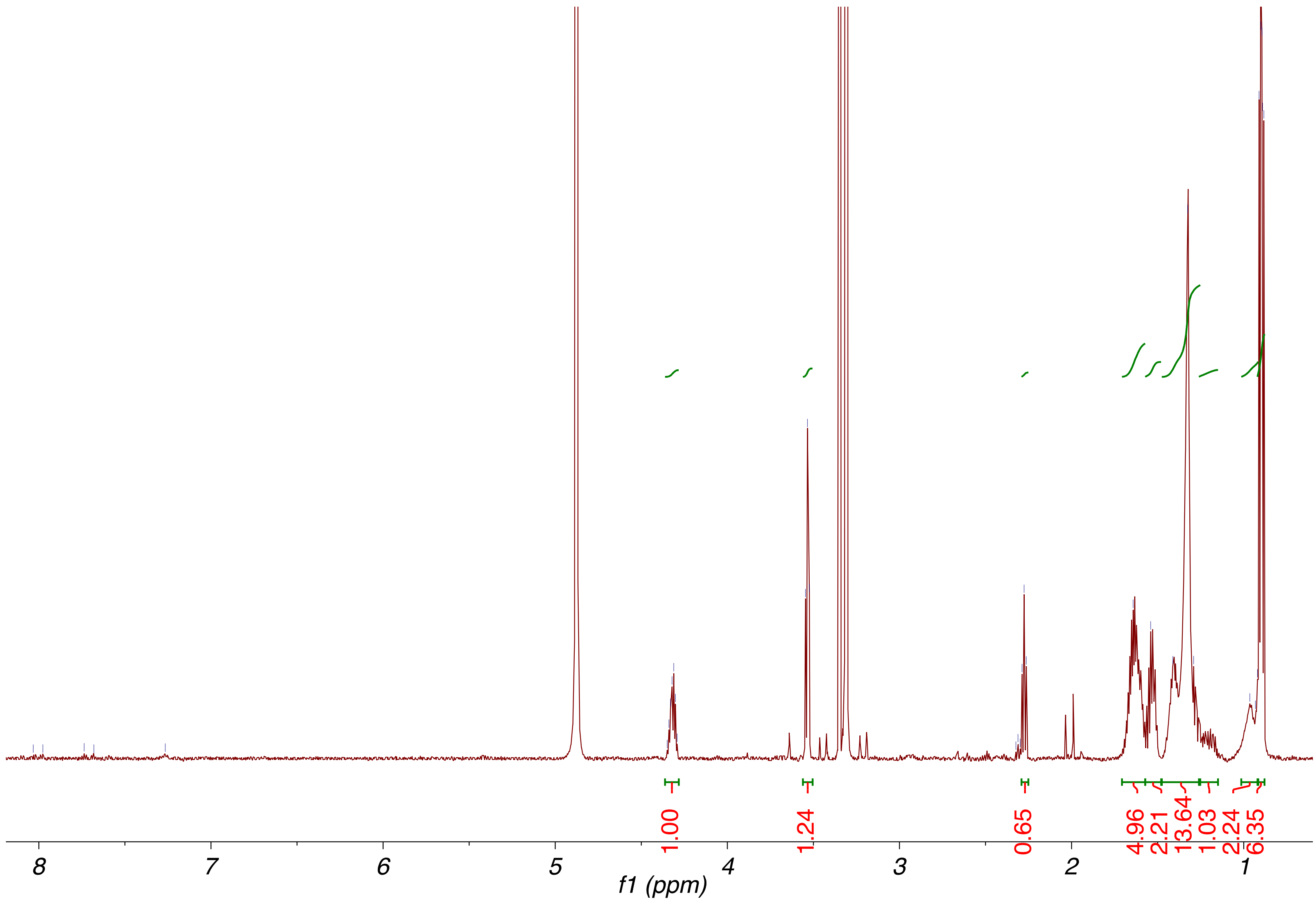
1.00
1.24
0.65
4.96
2.21
13.64
1.03
2.24
6.35
8
7
6
5
4
3
2
1
f1 (ppm)

dqfCOSY spectrum of active metabolome fraction containing sufac#1 and sufal#2 (600 MHz, methanol-d4).

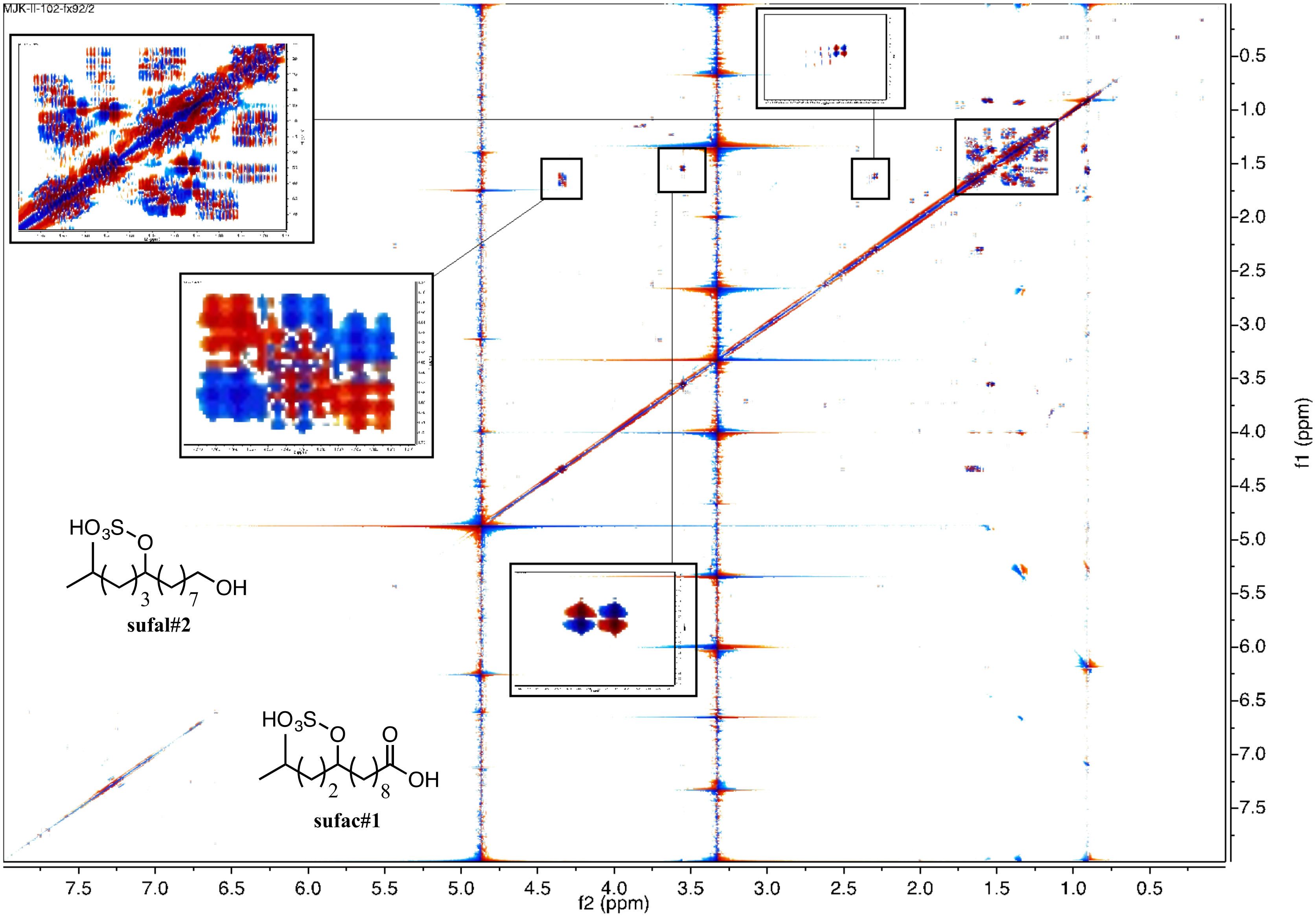

Coupled HSQC spectrum of active metabolome fraction containing sufac#1 and sufal#2 (600 MHz, methanol-d_4).

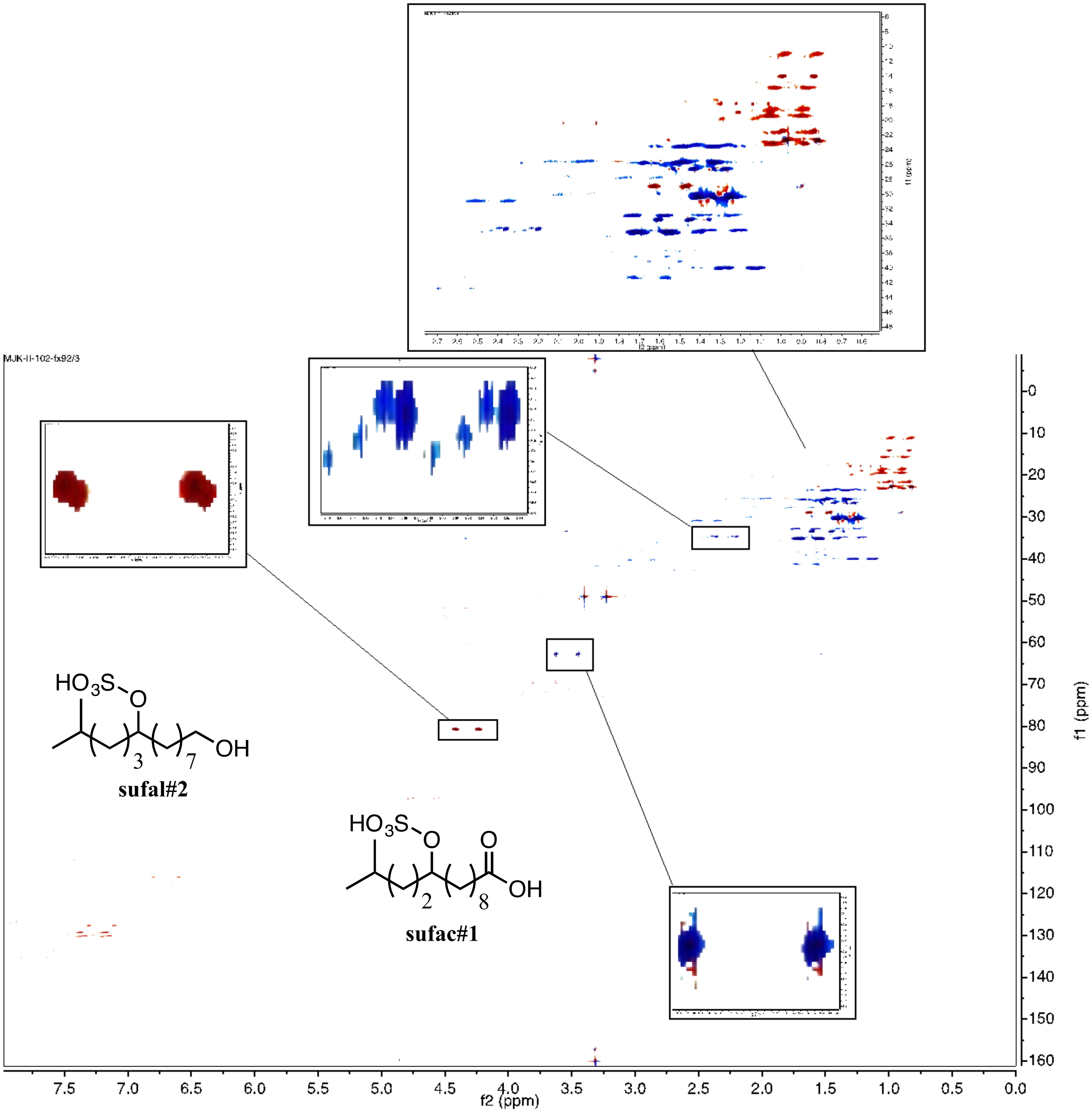
MJK-II-102-fx92/3
HO3S O OH 3 7
sufal#2
HO3S O O OH 2 8
sufac#1
f2 (ppm)
f1 (ppm)

Constant-time HMBC spectrum of active metabolome fraction containing sufac#1 and sufal#2 (600 MHz, methanol-d_4).

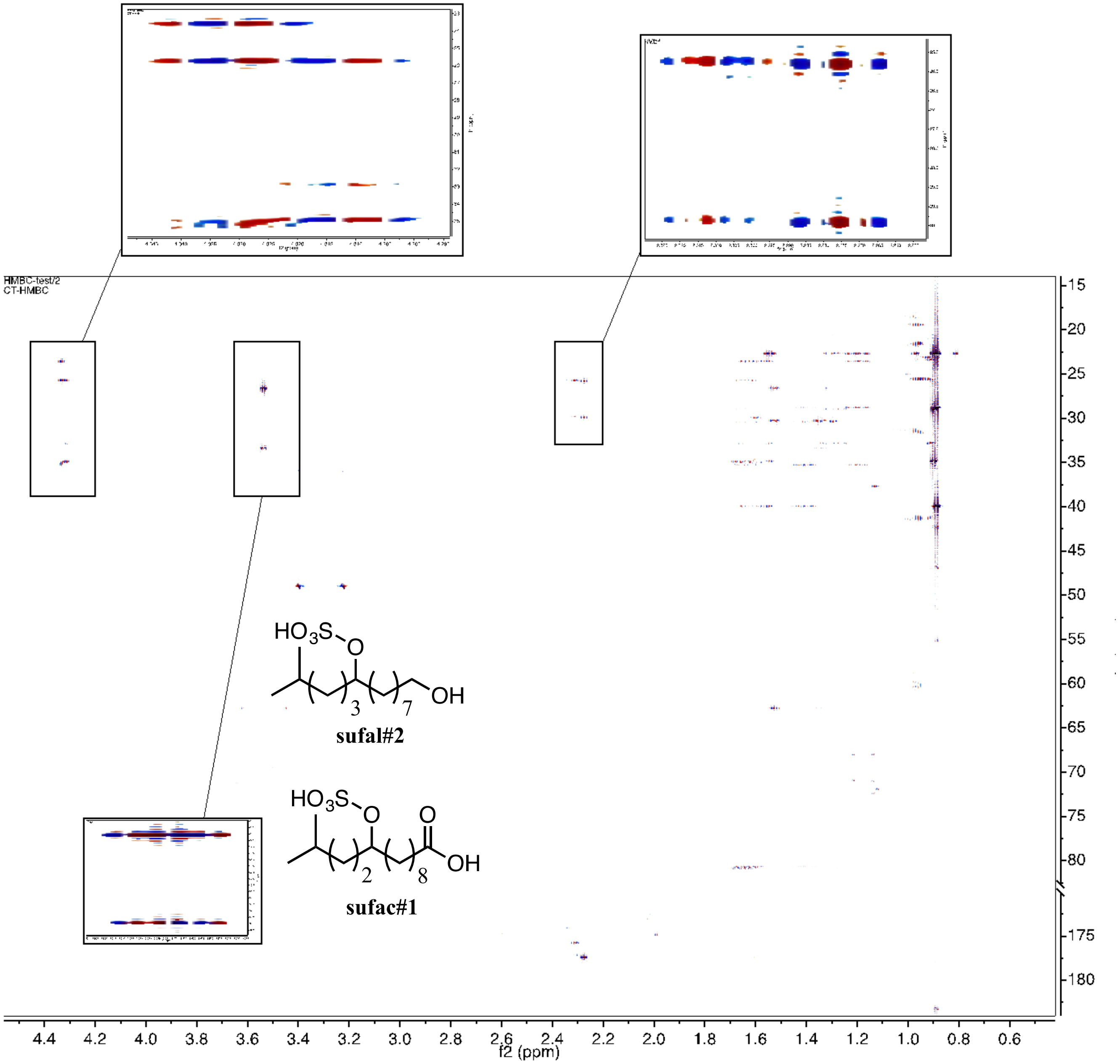
HMBC-test/2
CT-HMBC
f2 (ppm)
sufal#2
sufac#1

^{1}H NMR Spectrum (400 MHz, chloroform-*d*) of 2

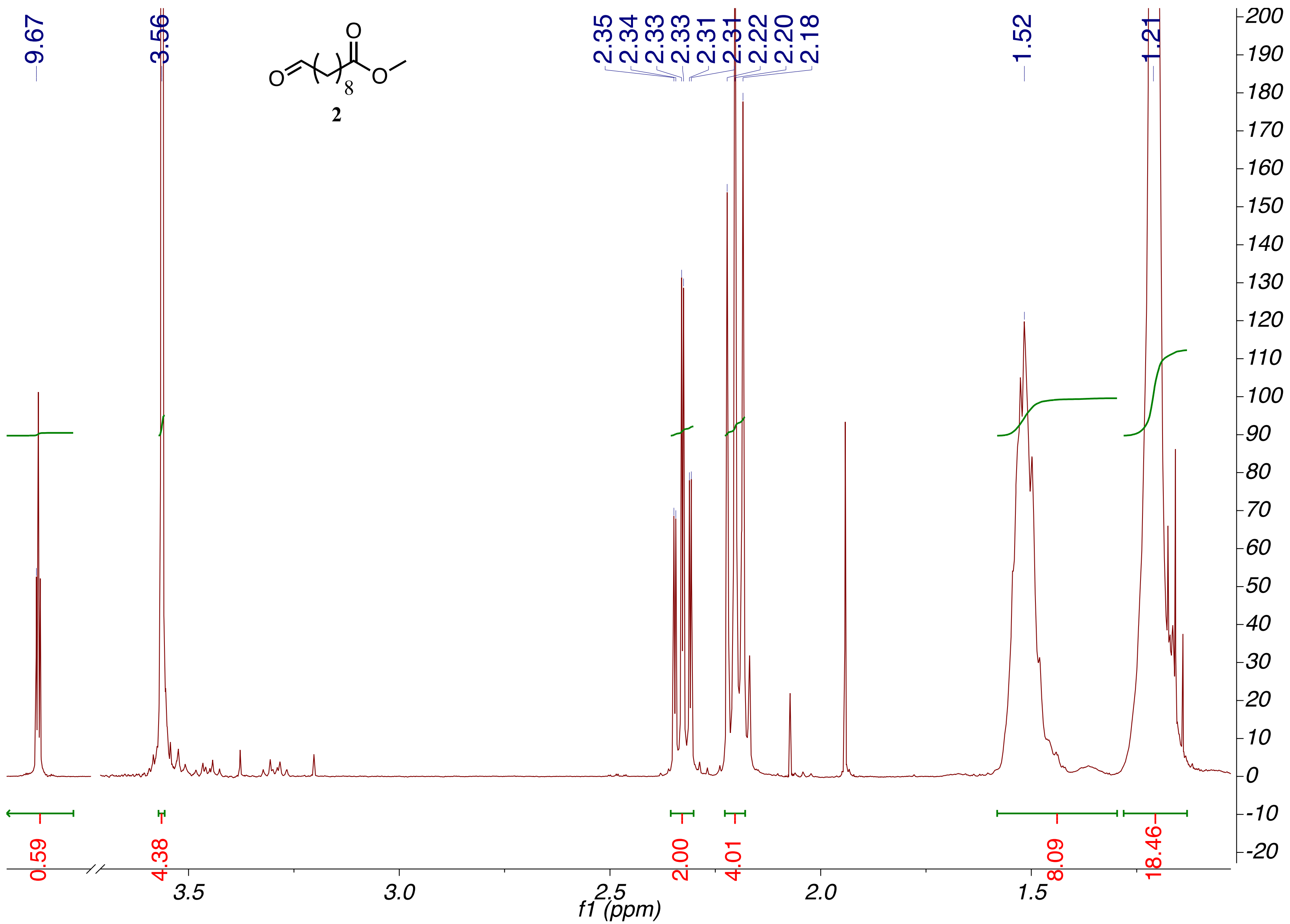

^{1}H NMR Spectrum (600 MHz, chloroform-*d*) of 3

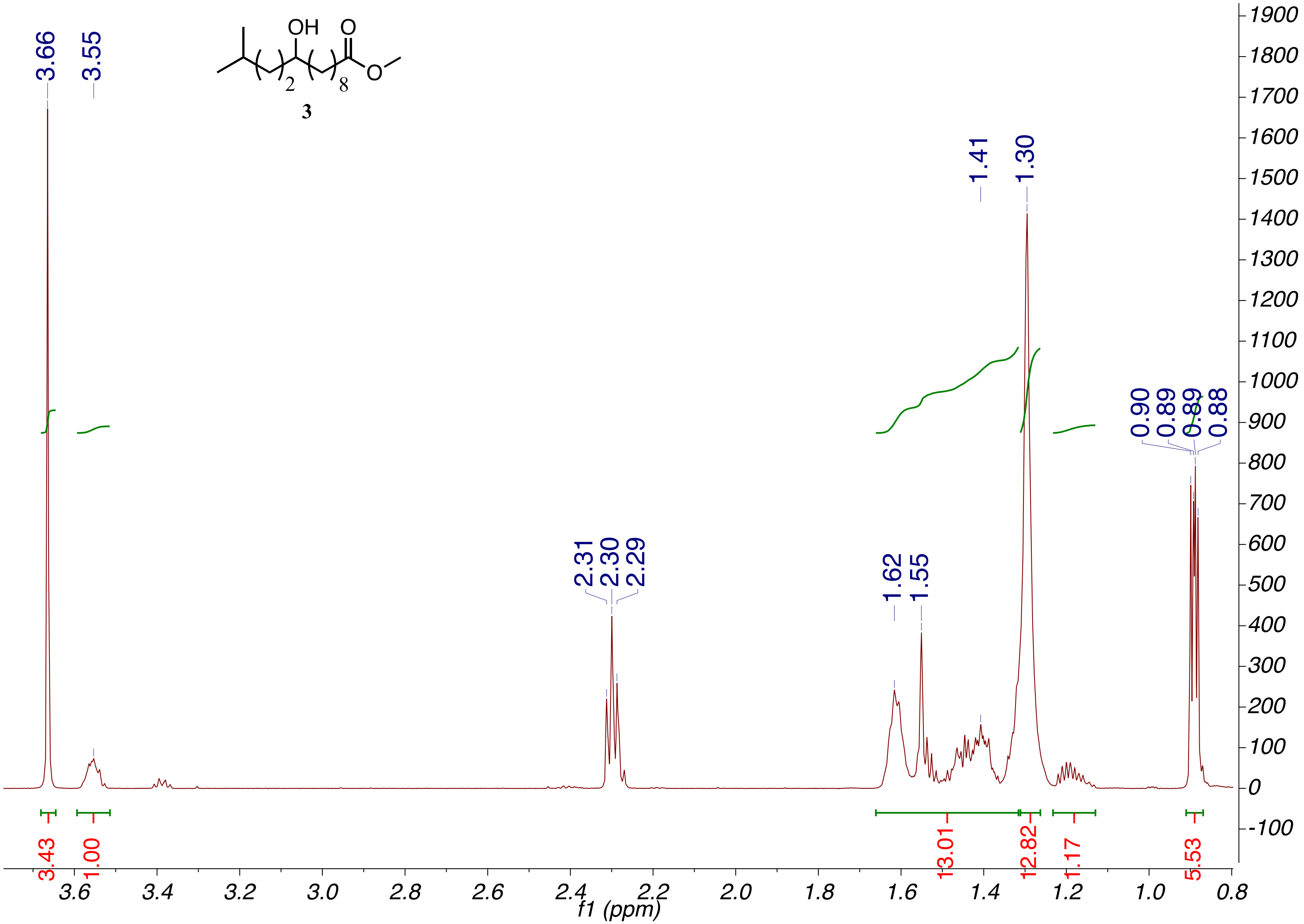

^{1}H NMR Spectrum (600 MHz, methanol-*d*₄) of 4

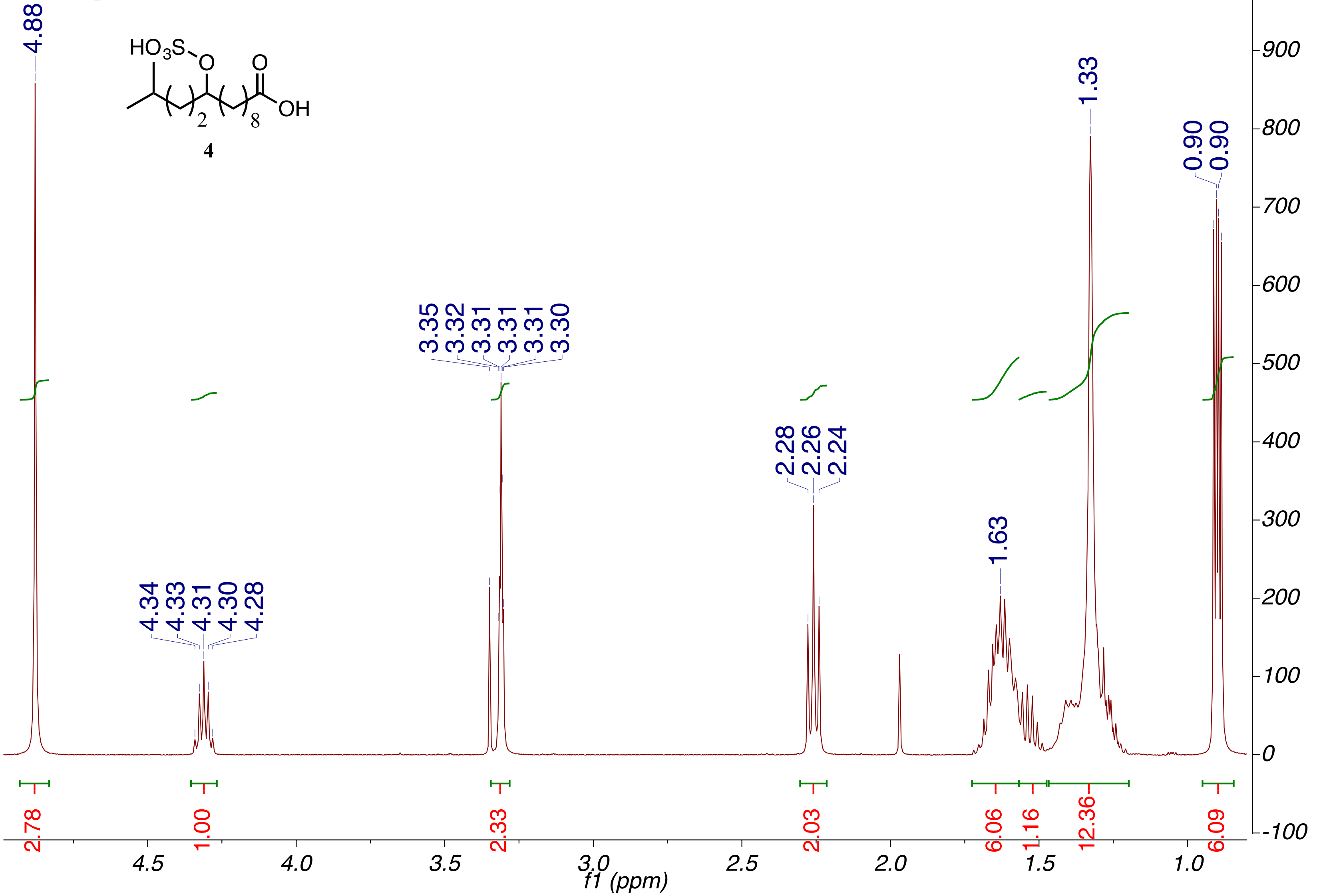

^{13}C NMR Spectrum (600 MHz, methanol-*d₄*) of 4

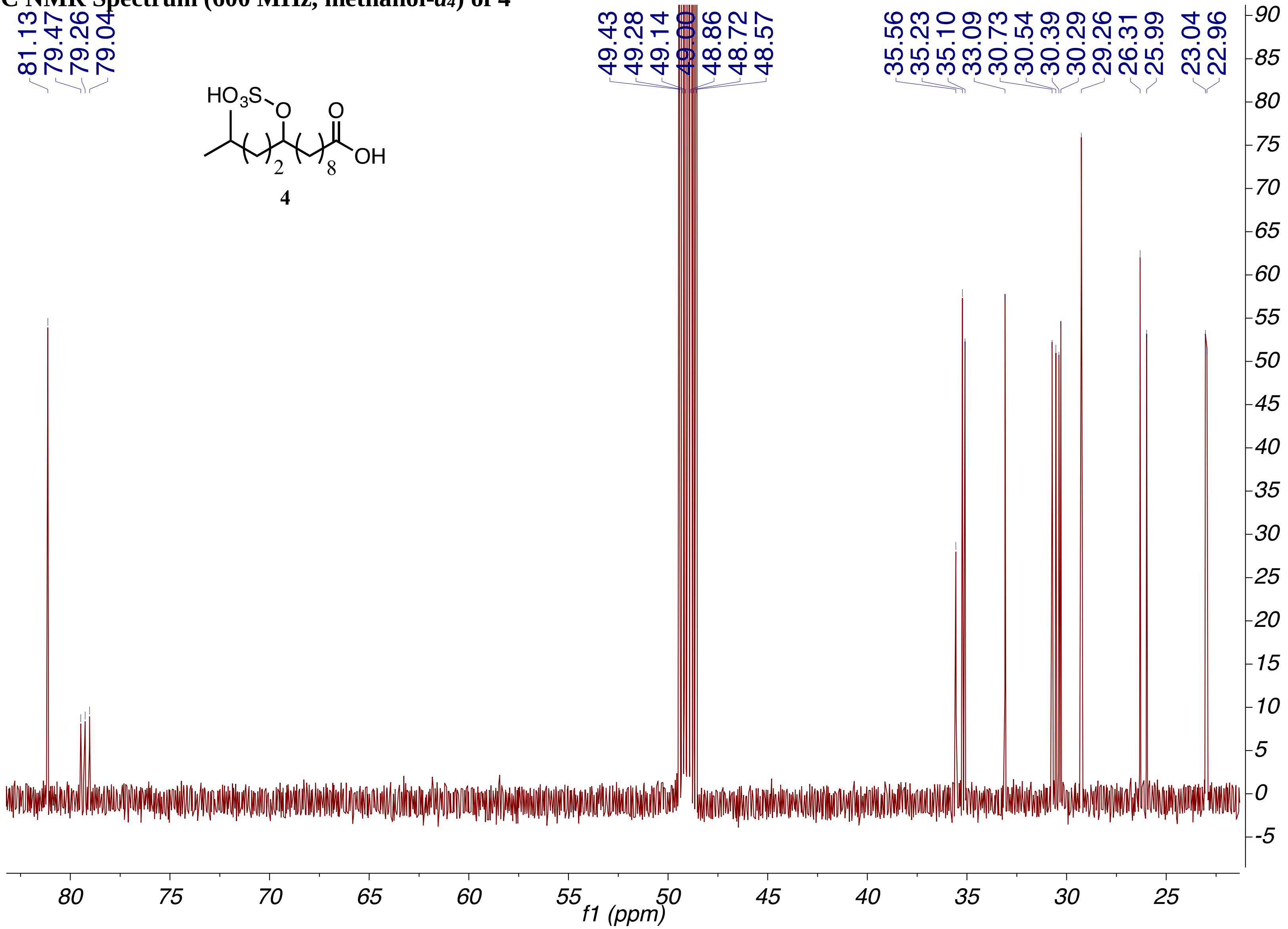

^{1}H NMR Spectrum (400 MHz, methanol-d_4) of 5

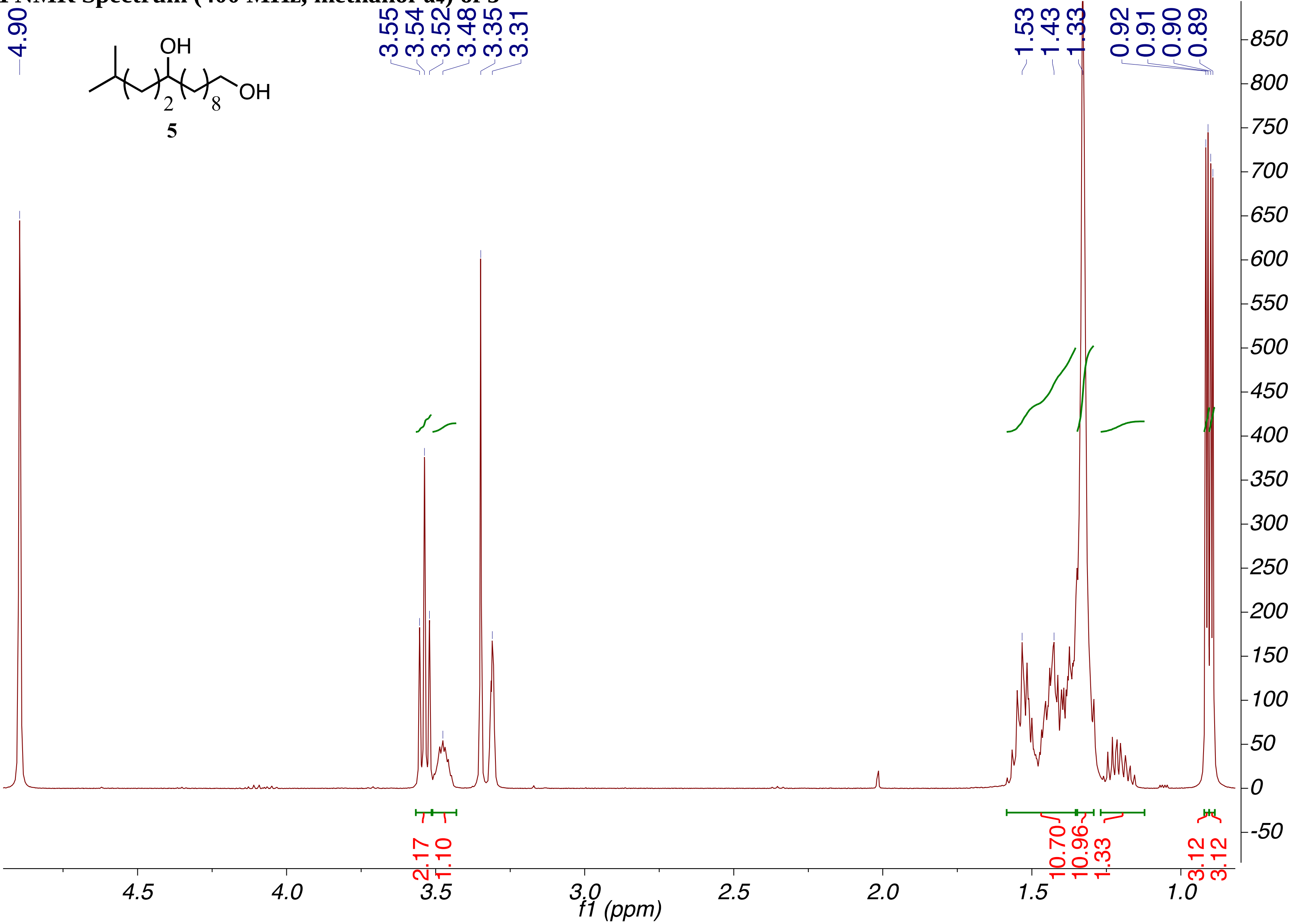

^{1}H NMR Spectrum (600 MHz, methanol-d_4) of 6

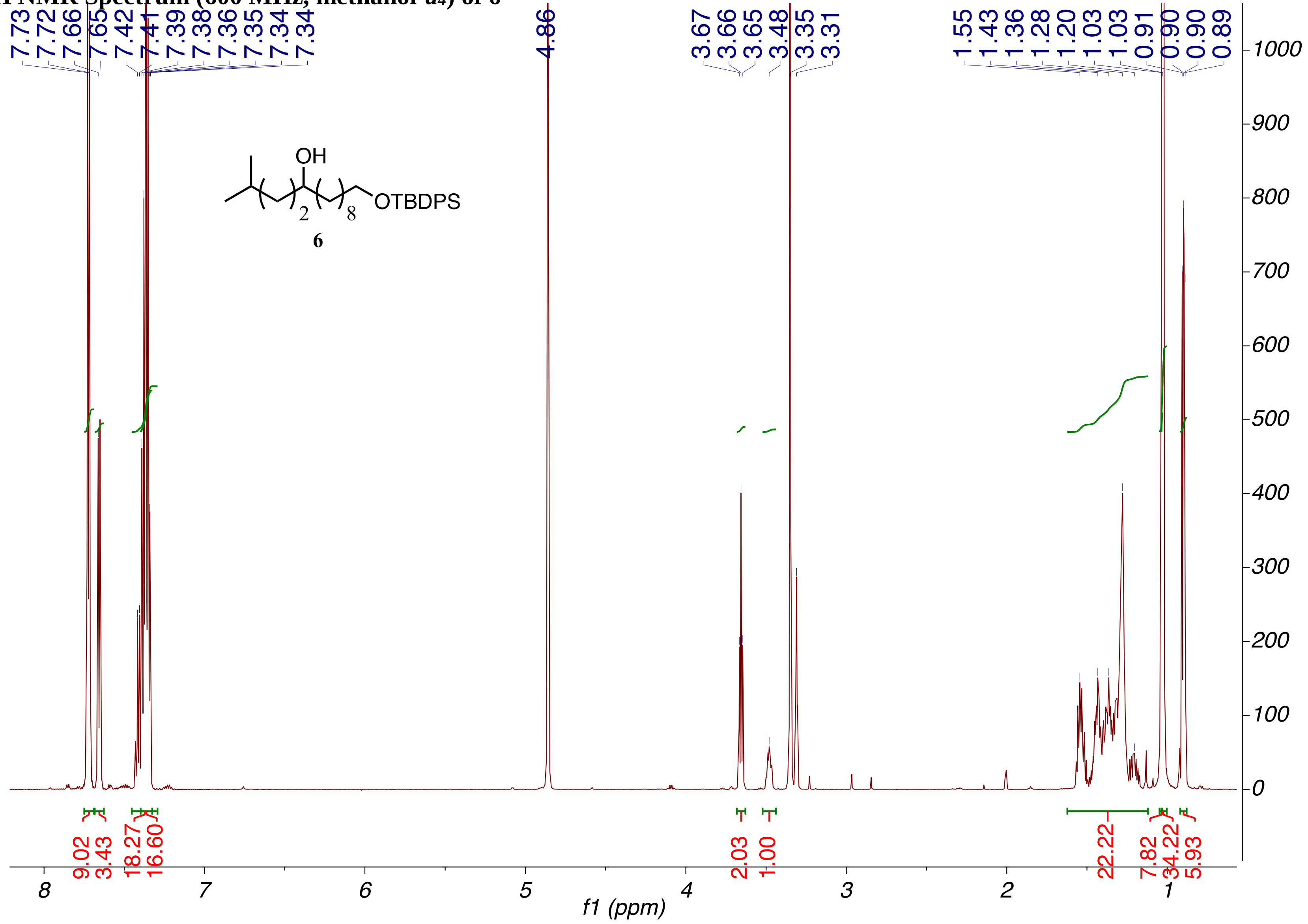

^{1}H NMR Spectrum (600 MHz, methanol-*d*$_4$) of 7

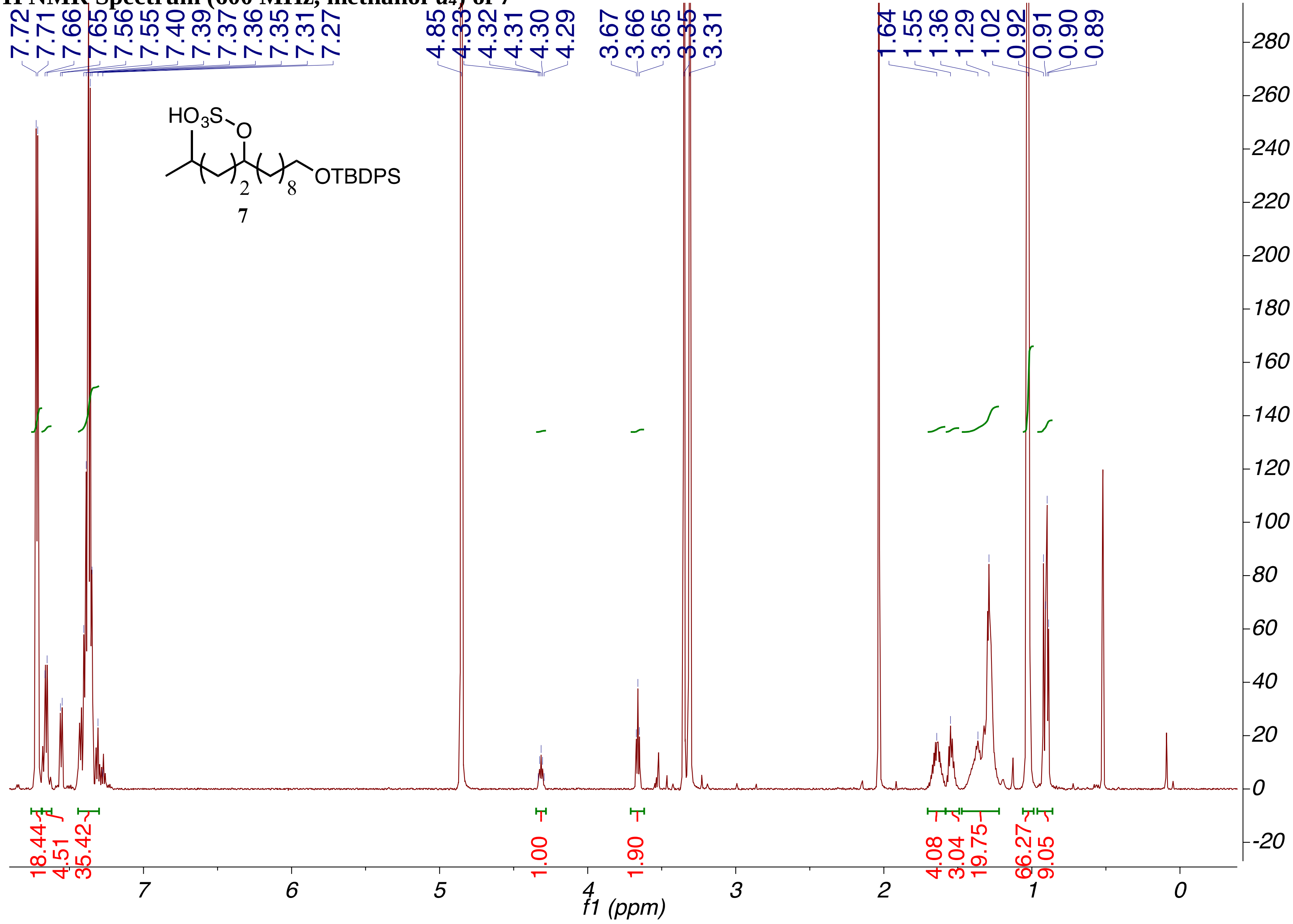

^{1}H NMR Spectrum (600 MHz, methanol-d_4) of 8

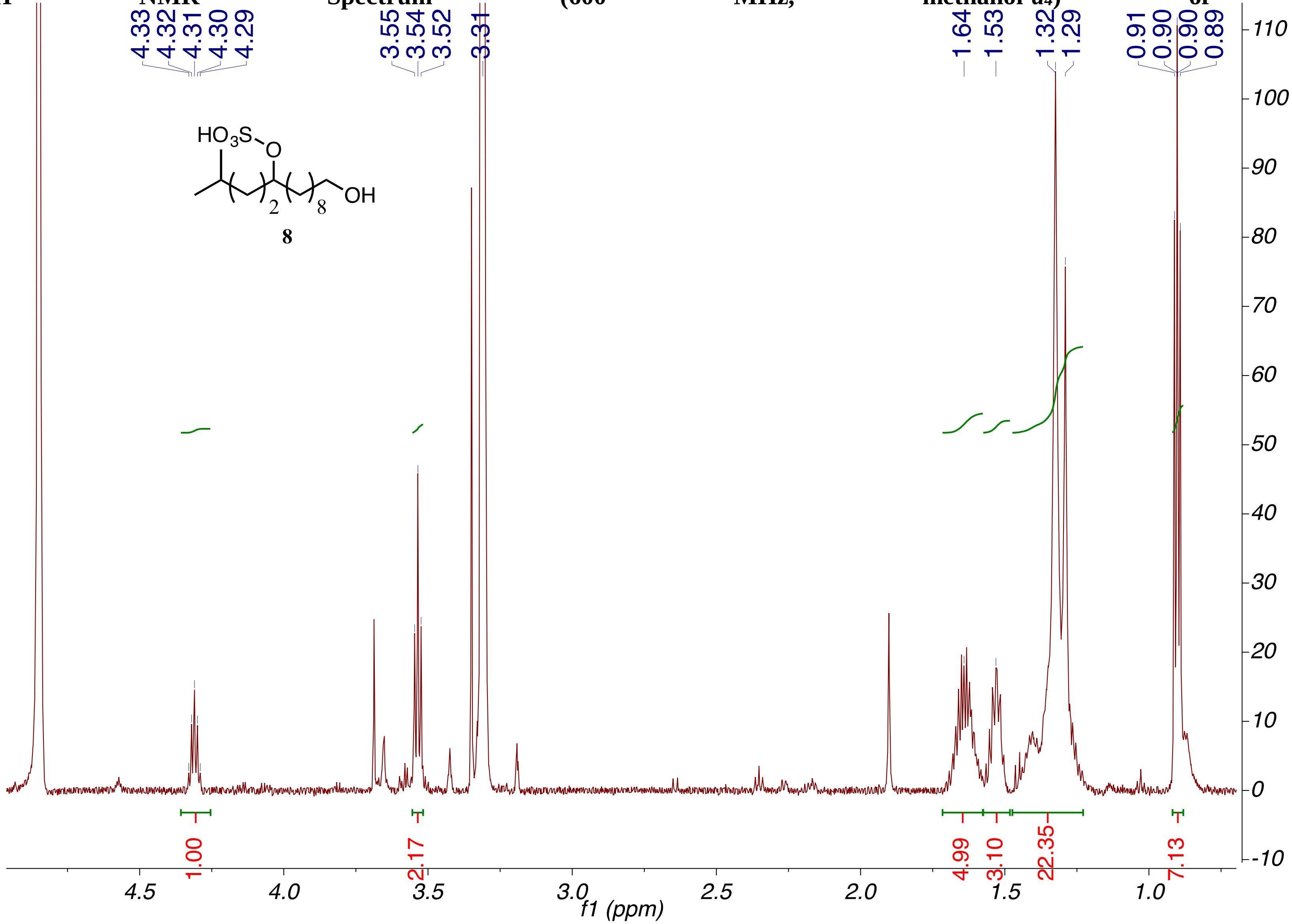

^{1}H NMR Spectrum of (600 MHz, chloroform-*d*) of 10

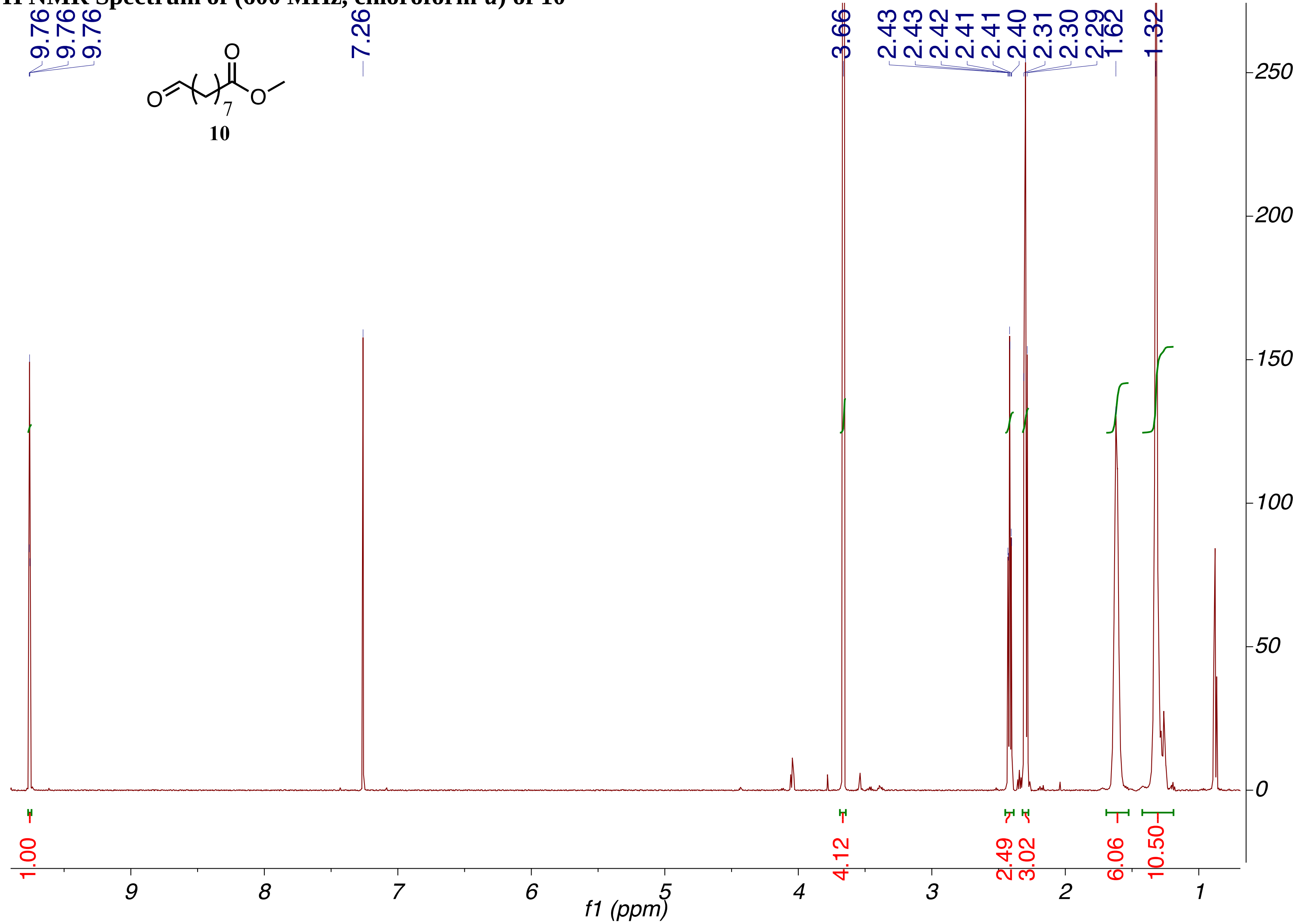

^{1}H NMR Spectrum (400 MHz, chloroform-*d*) of 11

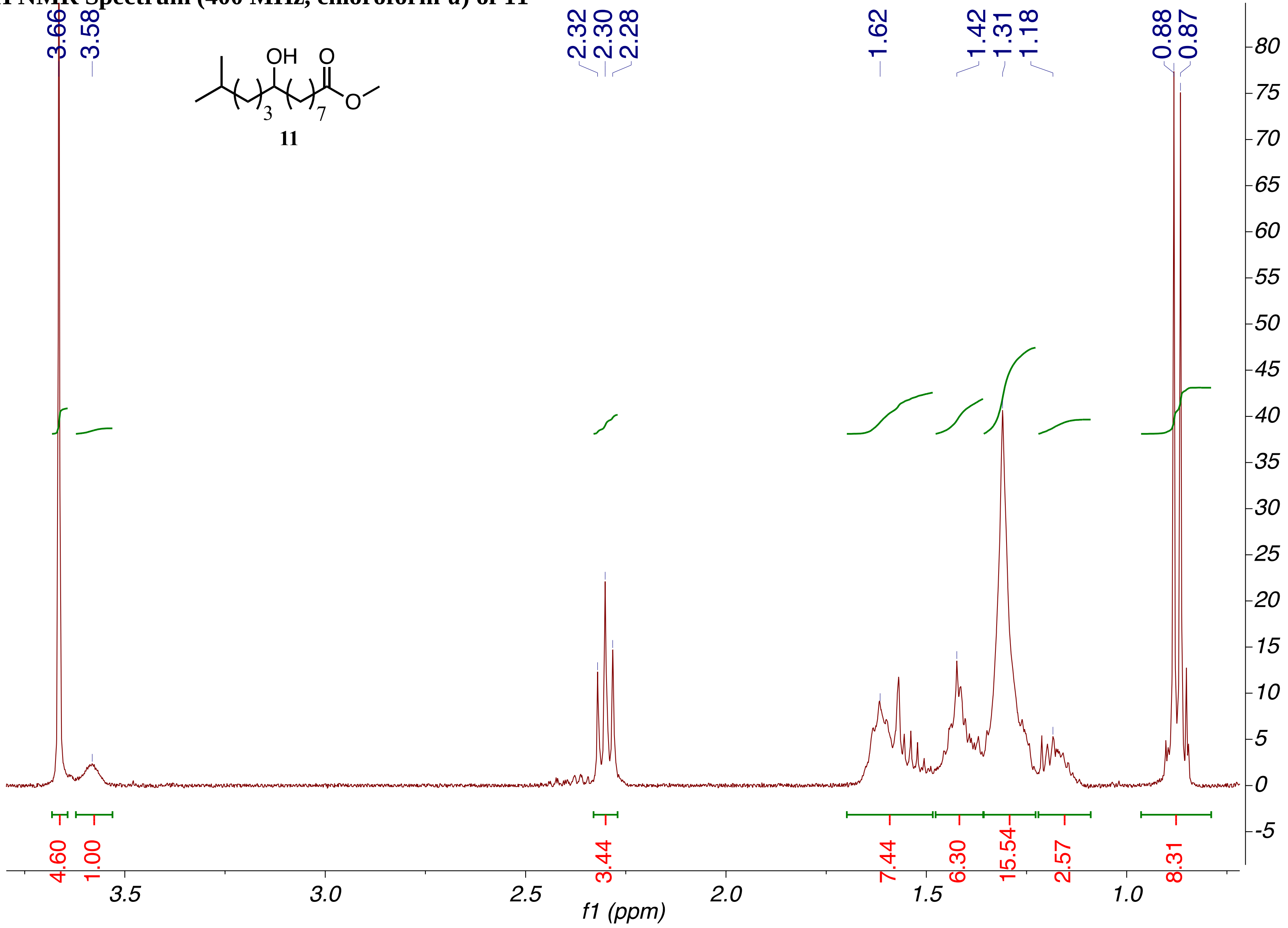

^{1}H NMR Spectrum (400 MHz, methanol-d_4) of 12

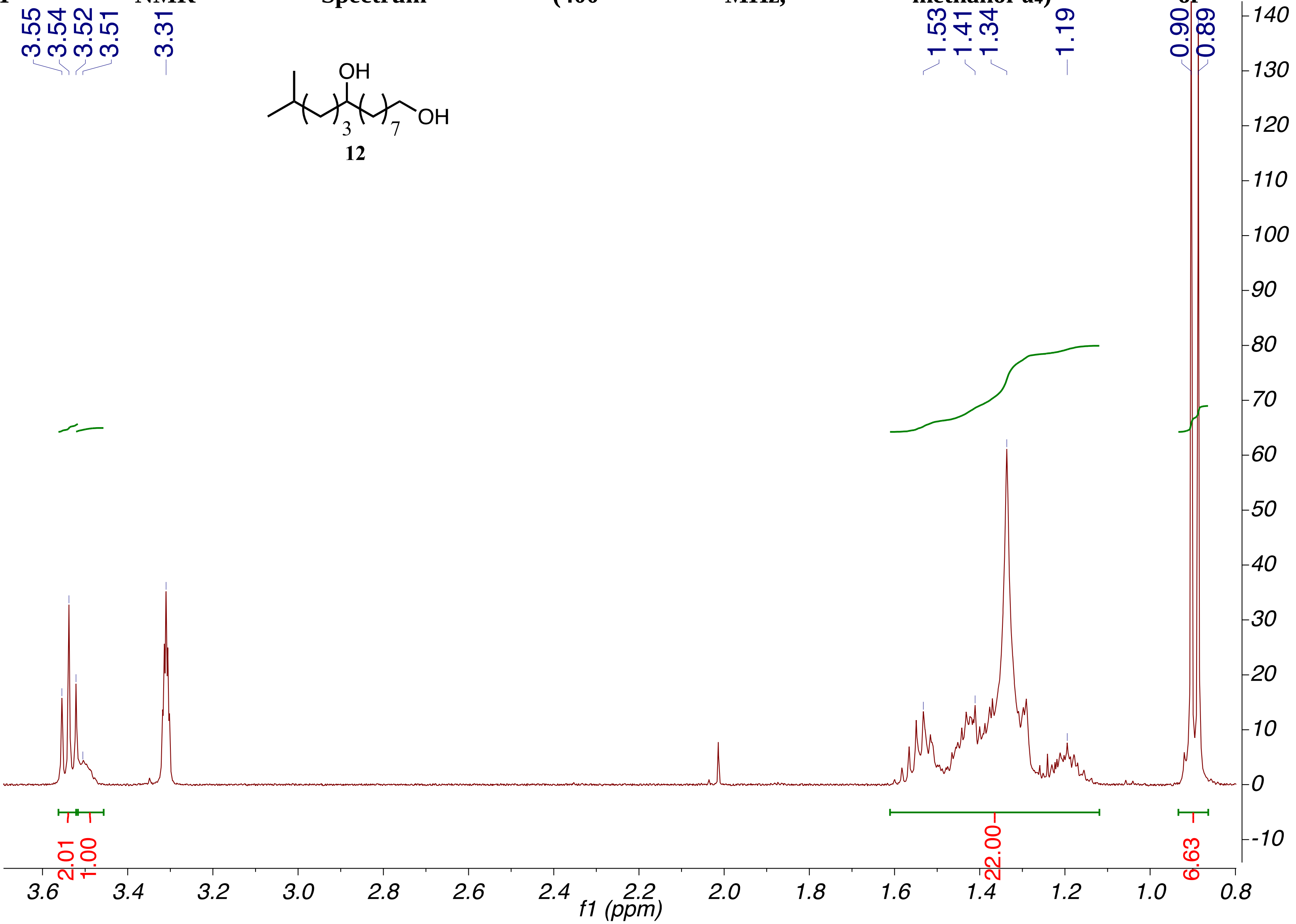

^{1}H NMR Spectrum (400 MHz, methanol-*d*$_4$) of 13

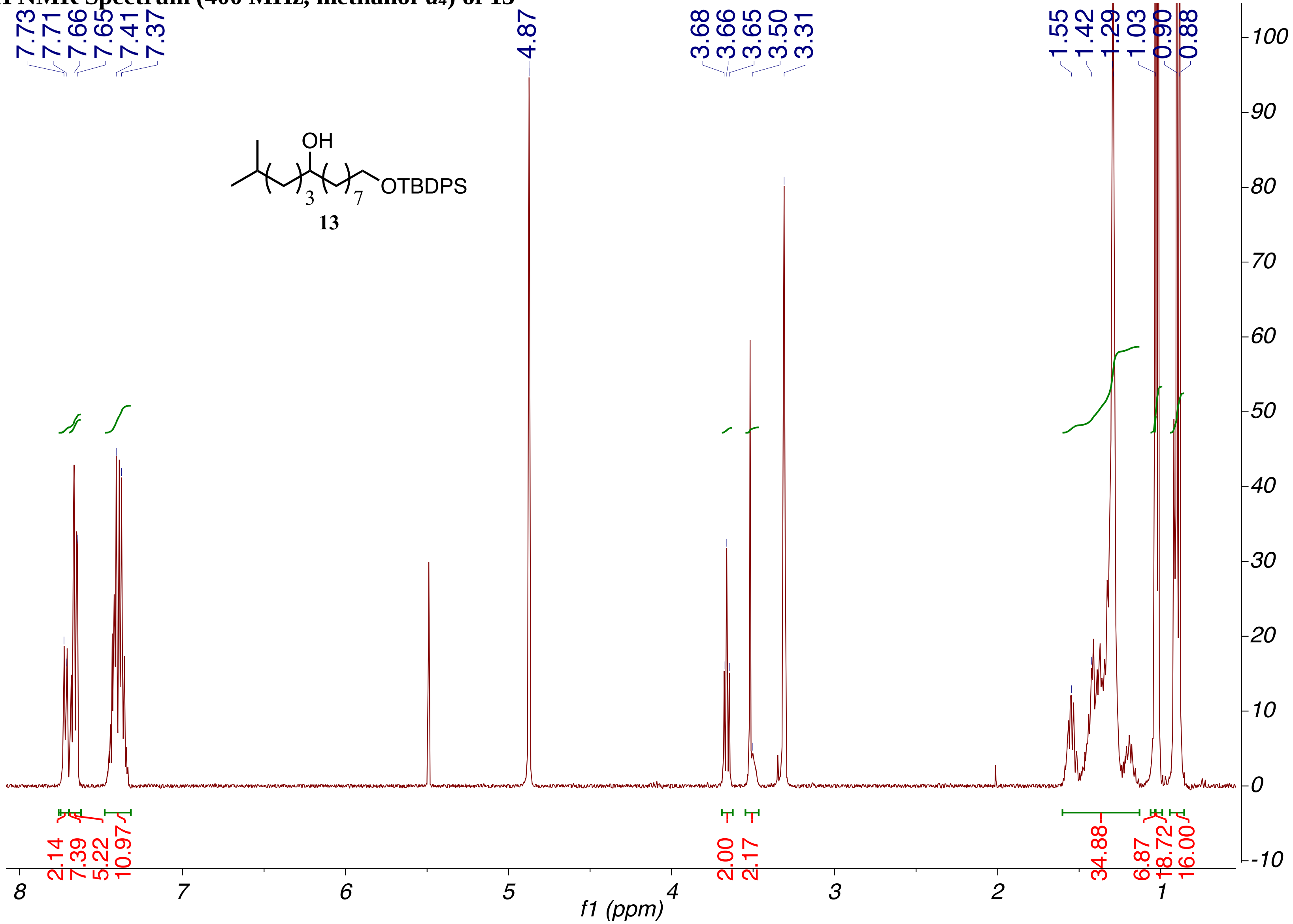

^{1}H NMR Spectrum (600 MHz, methanol-*d*$_4$) of 14

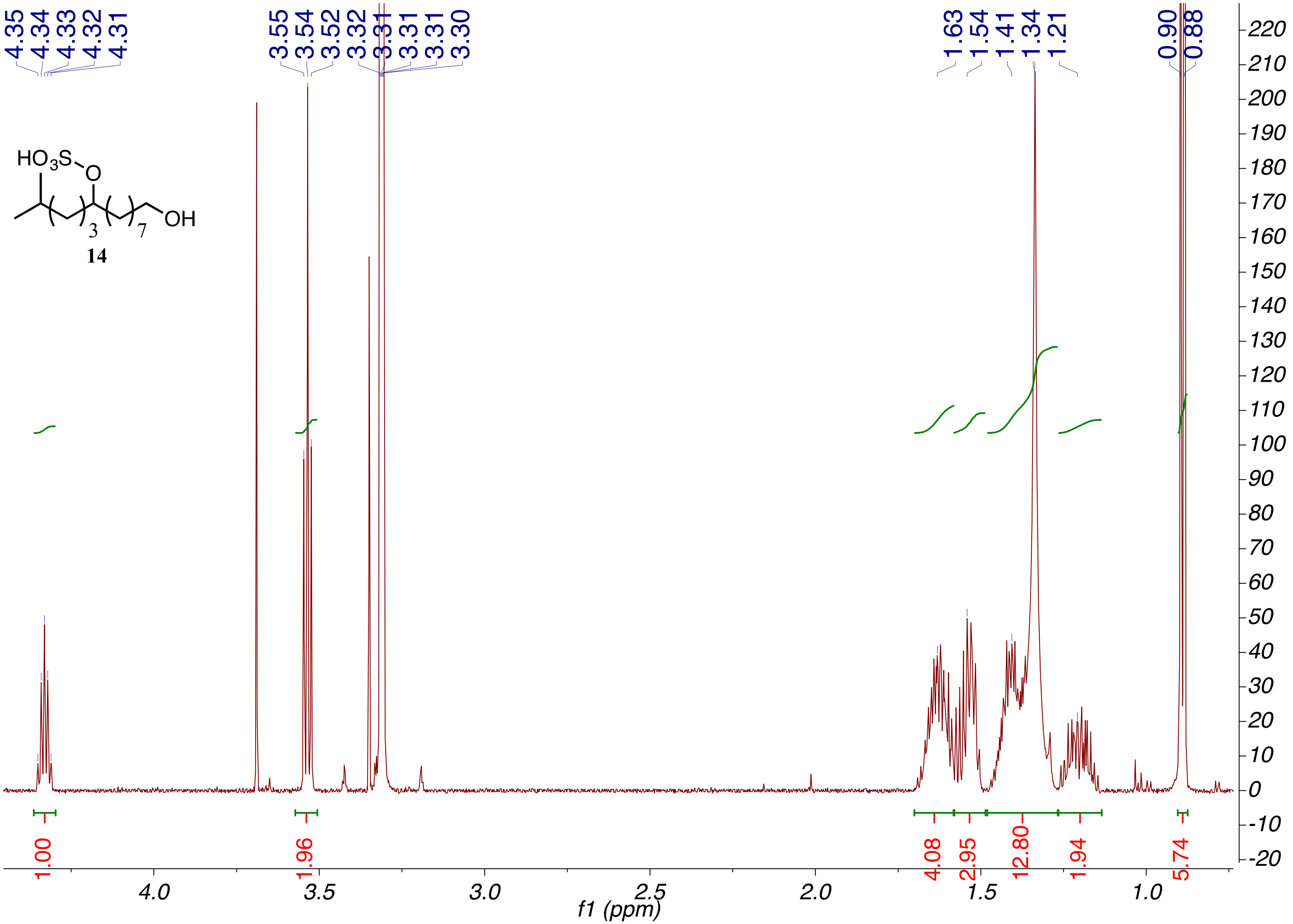

^{13}C NMR Spectrum (600 MHz, methanol-d_4) of 14

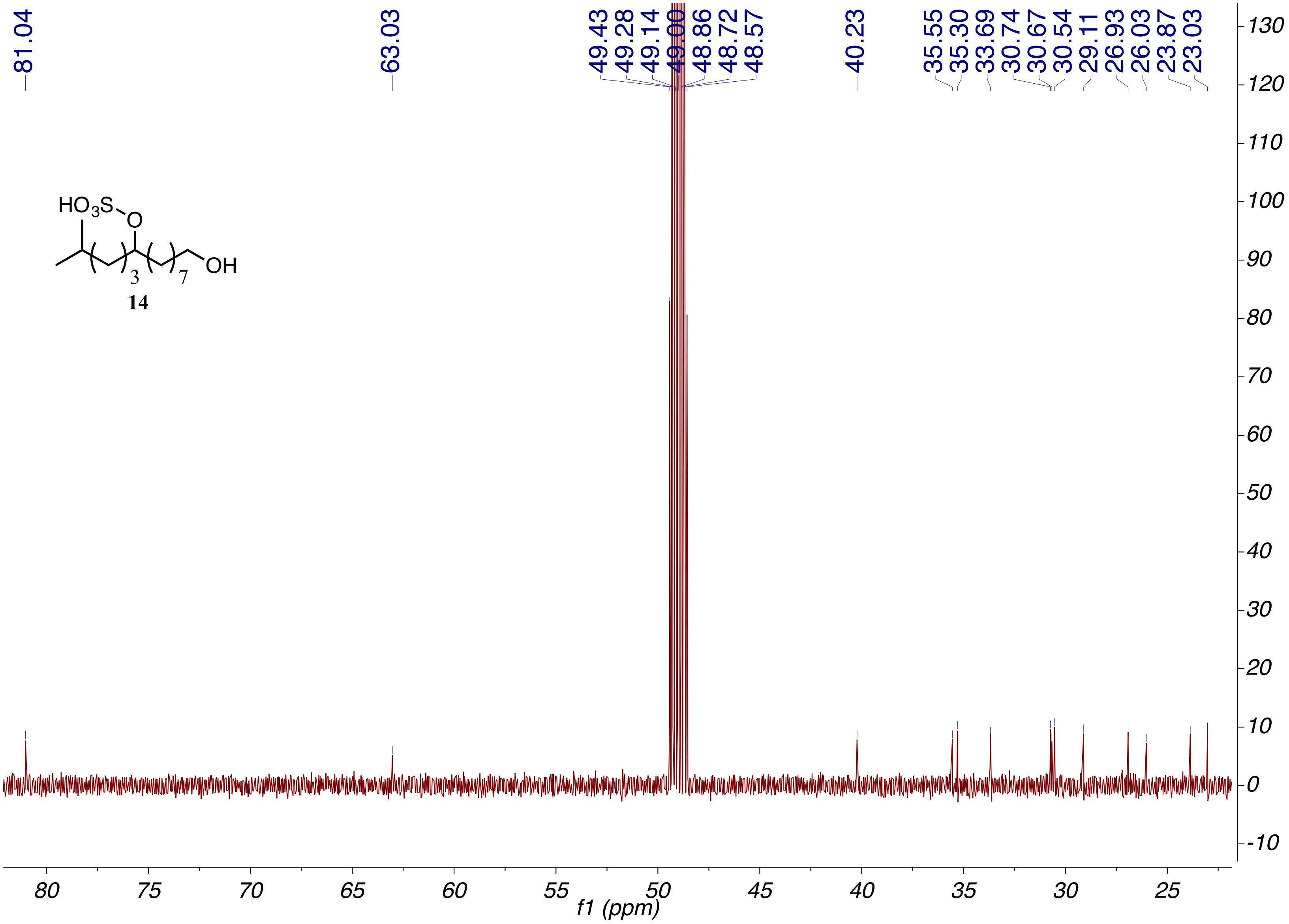

Supplement: Supplementary file 1 — Supplementary Information(PDF 2983 kb) [file 41467_2018_3333_MOESM1_ESM.pdf]
